# Supplementary figures and images for: Conjunctive spatial and self-motion codes are topographically organized in the GABAergic cells of the lateral septum
Source: PLoS Biol. 2021 Aug 30;19(8):e3001383. doi: 10.1371/journal.pbio.3001383 (PMC8432898; doi:10.1371/journal.pbio.3001383)

S1 Fig

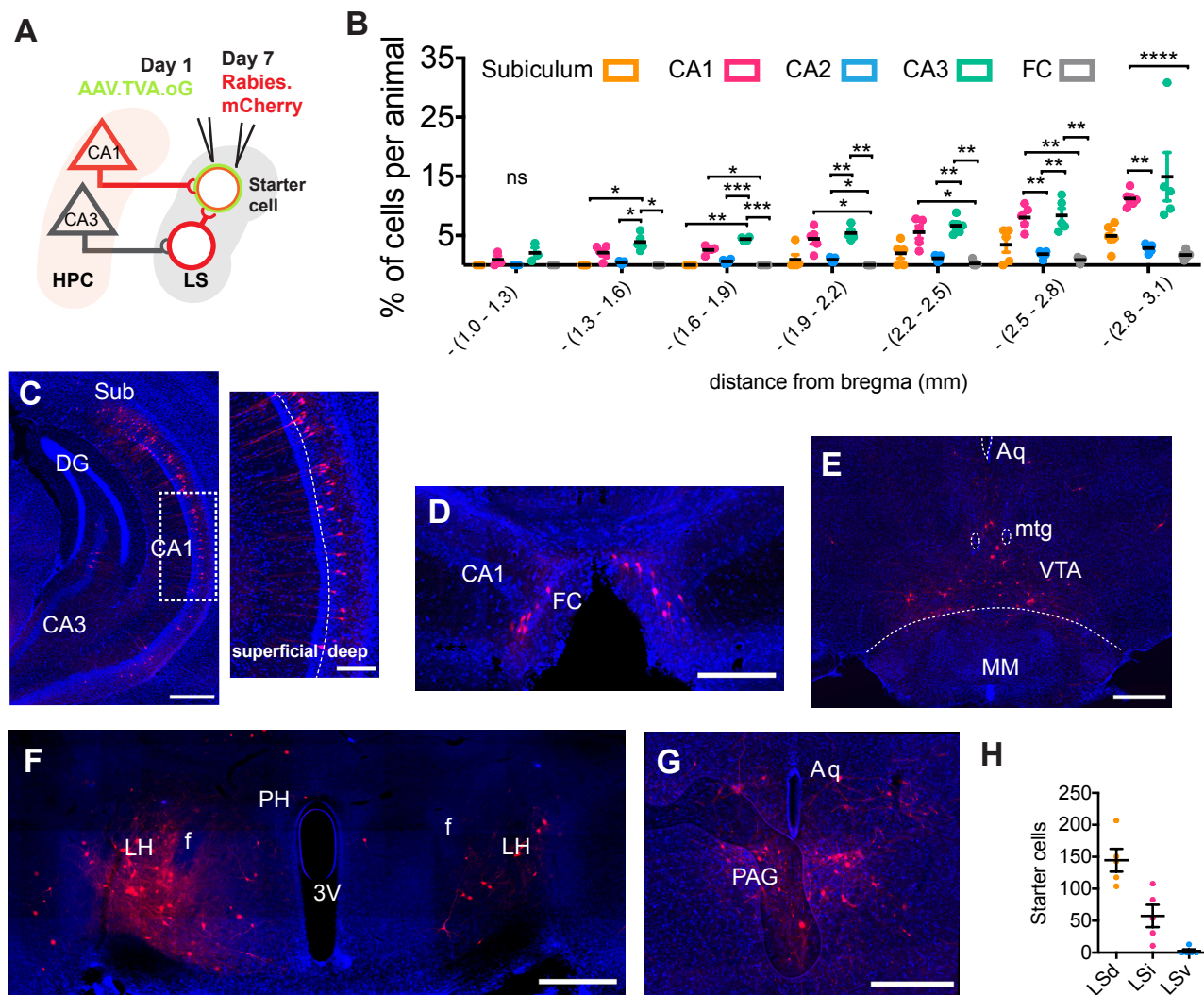

Supplement: S1 Fig — (A) Strategy for retrograde rabies tracing in LS. (B) Mean proportion of cells found in the hippocampus for each 300 μm coronal section; two-way RM ANOVA, F(24,126) = 2.738, p = 0.002, interaction effect, n = 5 mice. (C) Coronal section of ventral hippocampal region, showing retrogradely labeled cell bodies in subiculum, CA1, and CA3. (D) Retrogradely labeled cell bodies in the FC. (E) Retrogradely labeled cells in the VTA. (F) Bilateral labeling of the hypothalamic region. (G) Cell bodies in the periaqueductal gray. (H) Total number of starter cells for each LS subregion, ANOVA, F(2,12) = 24.61, p > 0.0001. Scale bars: C, left: 500 μm, right: 300 μm. D, 200 μm. E, 500 μm, F, 500 μm and G, 400 μm. *, p < 0.05, **, p < 0.01, ***, p < 0.001. Test used in B, one-way ANOVA. The underlying data can be found in S2 Data. Aq, aqueduct; f, fornix; DG, dentate gyrus; FC, fasciola cinereum; HPC, hippocampus; LH, lateral hypothalamus; LS, lateral septum; LSd, dorsal lateral septum; LSi, intermediate lateral septum; LSv, ventral lateral septum; MM, medial mammillary nucleus; mtg, mammillotegmental tract; ns, not significant; PAG, periaqueductal gray; PH, posterior hypothalamic area; Sub, subiculum; VTA, ventral tegmental area; 3V, third ventricle. (PDF) [file pbio.3001383.s003.pdf]

S2 Fig

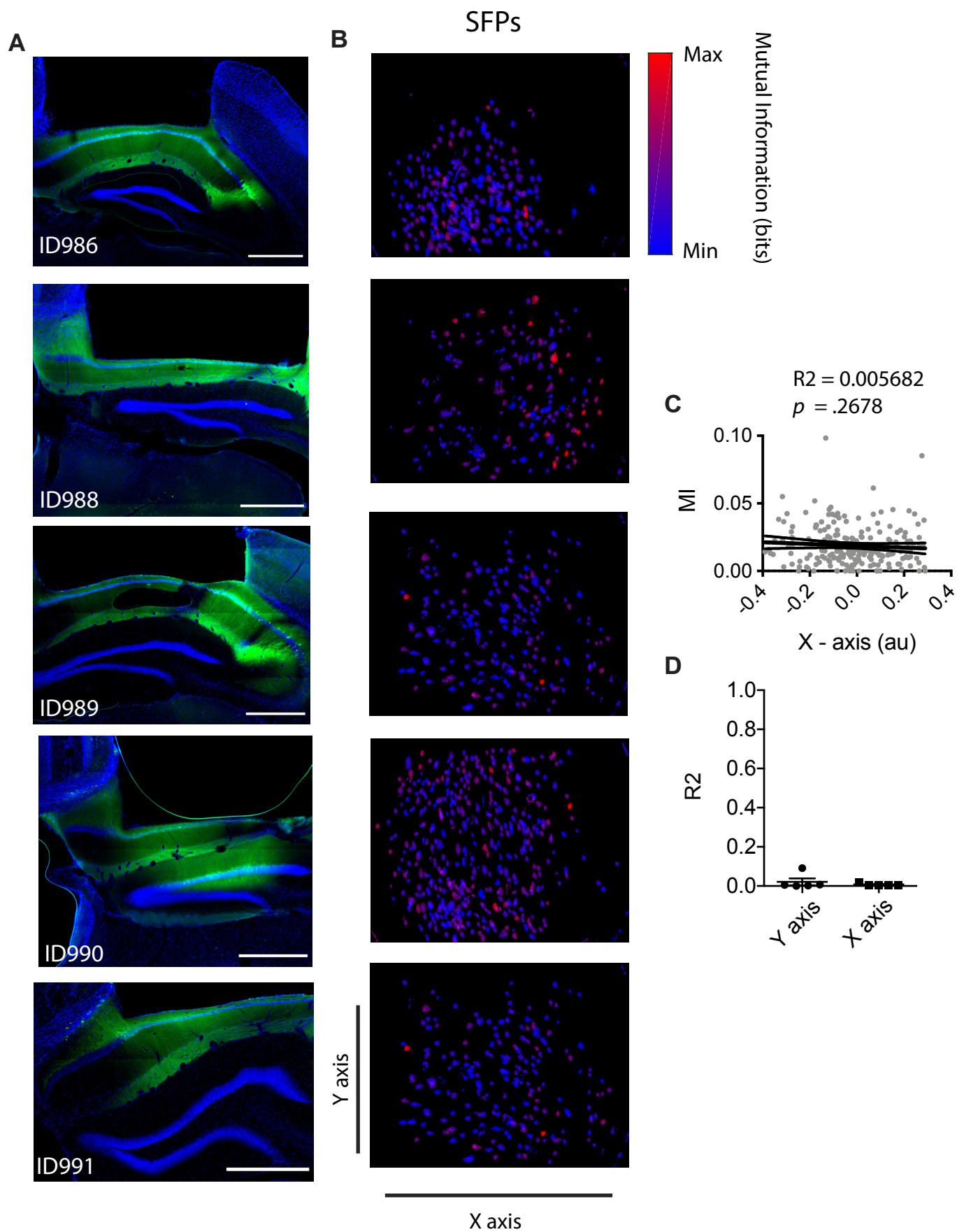

Supplement: S2 Fig — (A) Examples for CA1 animals included in 1D spatial navigation task, with Cre-dependent GCaMP6f (green) in a CaMKIIα-Cre mouse with DAPI counterstaining (blue). (B) Corresponding SFPs from extracted cells, color coded for minimum to maximum MI value. (C) Example of approach to compute the correlation between MI and position on the x-axis, linear regression R2 = 0.005682, p = 2678. (D) Summary of all R2 values for n = 5 mice. The underlying data can be found in S2 Data. MI, mutual information; SPF, spatial footprint. (PDF) [file pbio.3001383.s004.pdf]

S3 Fig

**A**

trans-hippocampal implant

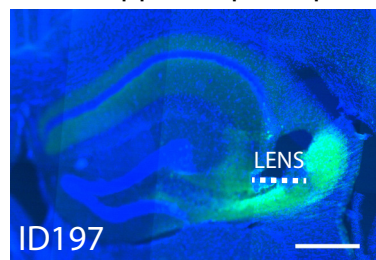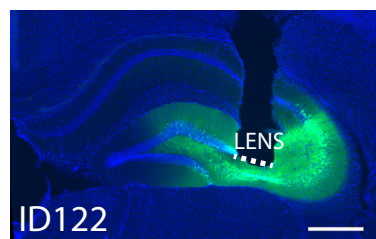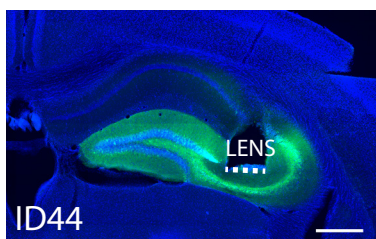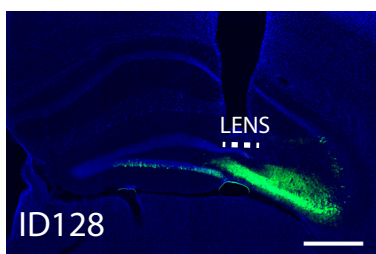

extra-hippocampal implant

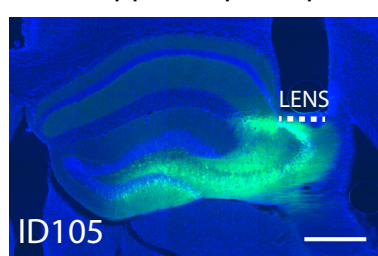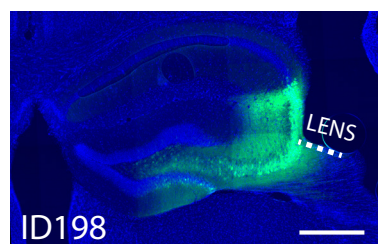

**B**

anterior - posterior axis

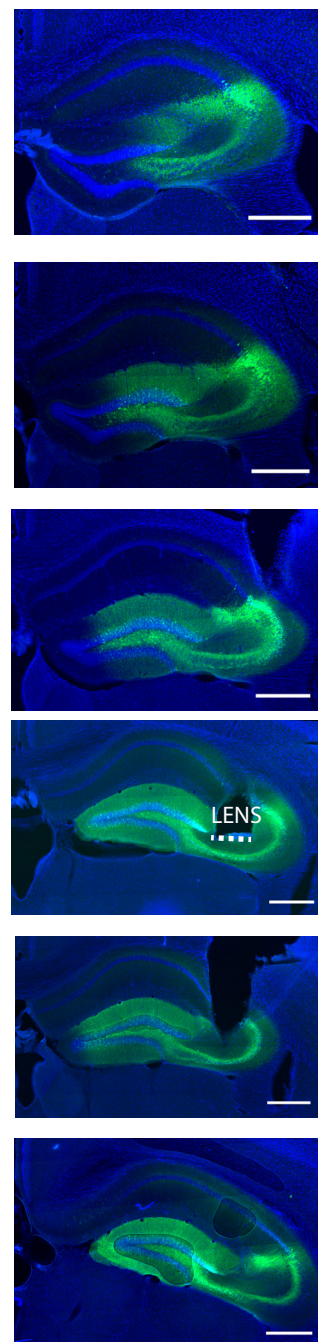

**C**

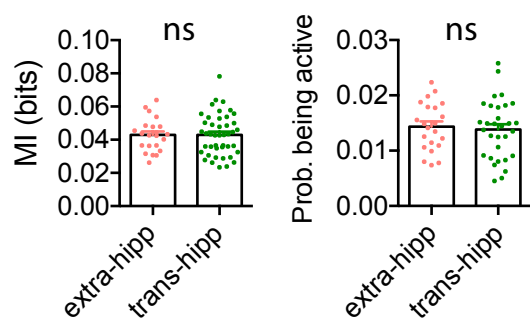

Supplement: S3 Fig — (A) Coronal section of dorsal hippocampal region, showing location of GRIN lens implant. Left: animals implanted trans-hippocampally. Right: animals implanted with an extrahippocampal approach. (B) Coronal sections from anterior to progressively more posterior regions of the hippocampus, showing extent of damage for a 500-μm lens implant. (C) Comparison between MI and probability being active for spatially modulated cells recorded in animals with trans-hippocampal vs. extrahippocampal implants (MI: unpaired t test, t(64) = 0.0367, p = 0.9708; P(A): unpaired t test, t(51) = 0.3908, p = 6875; extra-hipp: n = 22 cells from 2 animals, trans-hipp: n = 44 cells from 3 animals). The underlying data can be found in S2 Data. GRIN, gradient refractive index; MI, mutual information; ns, not significant. (PDF) [file pbio.3001383.s005.pdf]

S4 Fig

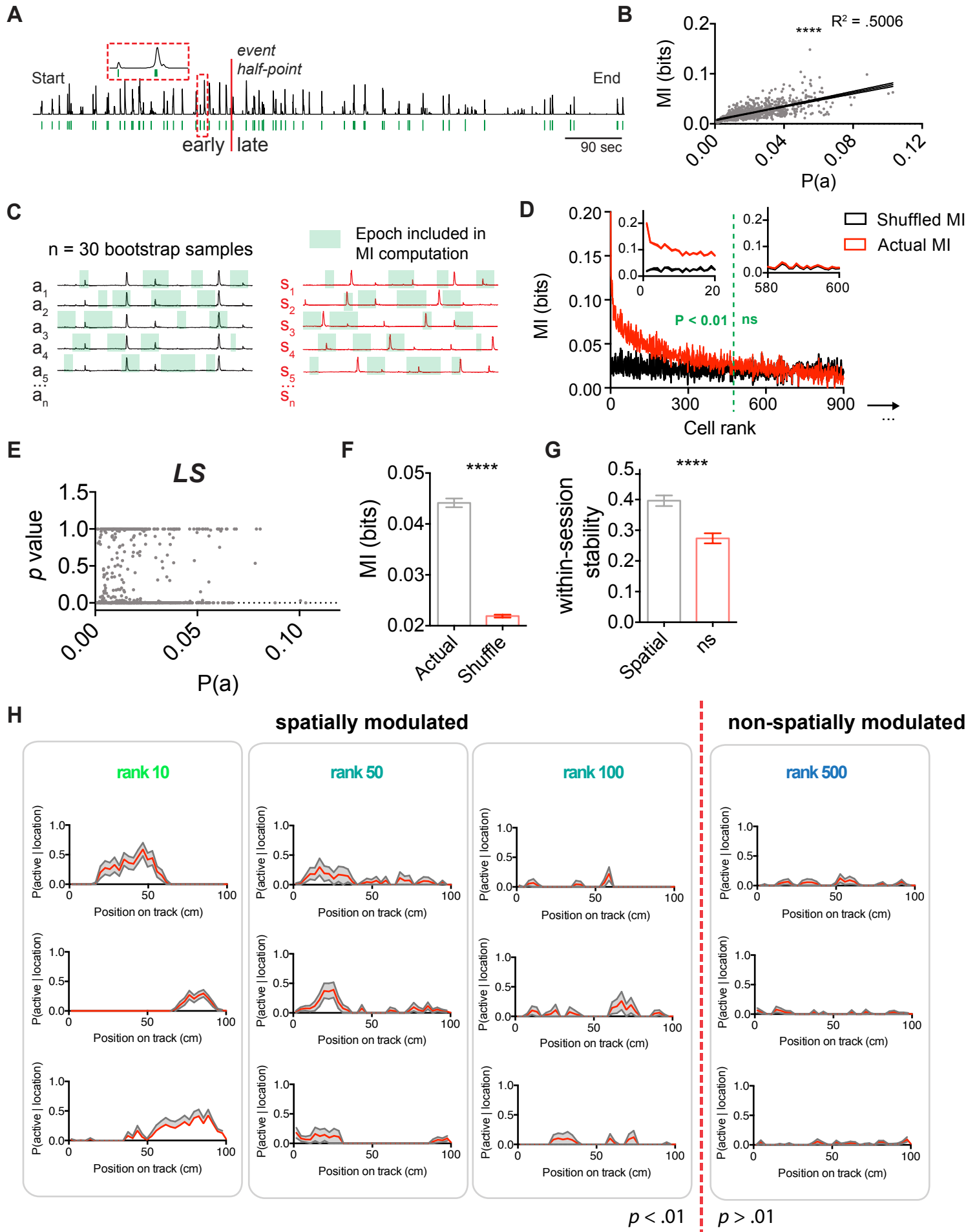

Supplement: S4 Fig — (A) Example of raw calcium fluorescence trace (top) and binarized events (green, bottom). Trace is divided into early and late recording epochs using 50% of total calcium (using the area under the curve). Inset: zoomed calcium trace with binarized traces. (B) Linear correlation between MI and probability being active (linear regression, R2 = 0.5006, p < 0.0001, n = 1,030 cells from n = 15 mice). (C) Method for computing the MI and 95% confidence interval from bootstrapped samples (left) and 30× shuffled surrogates (right). (D) MI computed from actual traces (black) and shuffled traces (red), sorted by the magnitude of the difference between these values, two-way ANOVA, F(1029,59740) = 126.3, p < 0.0001 for interaction effect. Left inset: zoomed version first 20 cells. Right inset: zoomed version for 20 not significant cells. (E) Scatterplot for significance level for spatial modulation and probability being active (linear regression, R2 = 0.0145, p = 0.007, n = 1,030 cells, n = 15 mice). (F) Group averages of actual (gray) vs. shuffled (red) MI for spatially modulated LS cells recorded on linear track (Mann–Whitney test, U = 116,394, p < 0.0001). (G) LS group averages of spatial cells (gray) vs. nonspatial cells (red) for split half stability (Mann–Whitney test, U = 17,743, p < 0.0011). (H) Examples of significantly spatially modulated cells representative for each rank, as ranked according to panel C. *, p < 0.05, ****, p < 0.0001. The underlying data can be found in S2 Data. LS, lateral septum; MI, mutual information. (PDF) [file pbio.3001383.s006.pdf]

S5 Fig

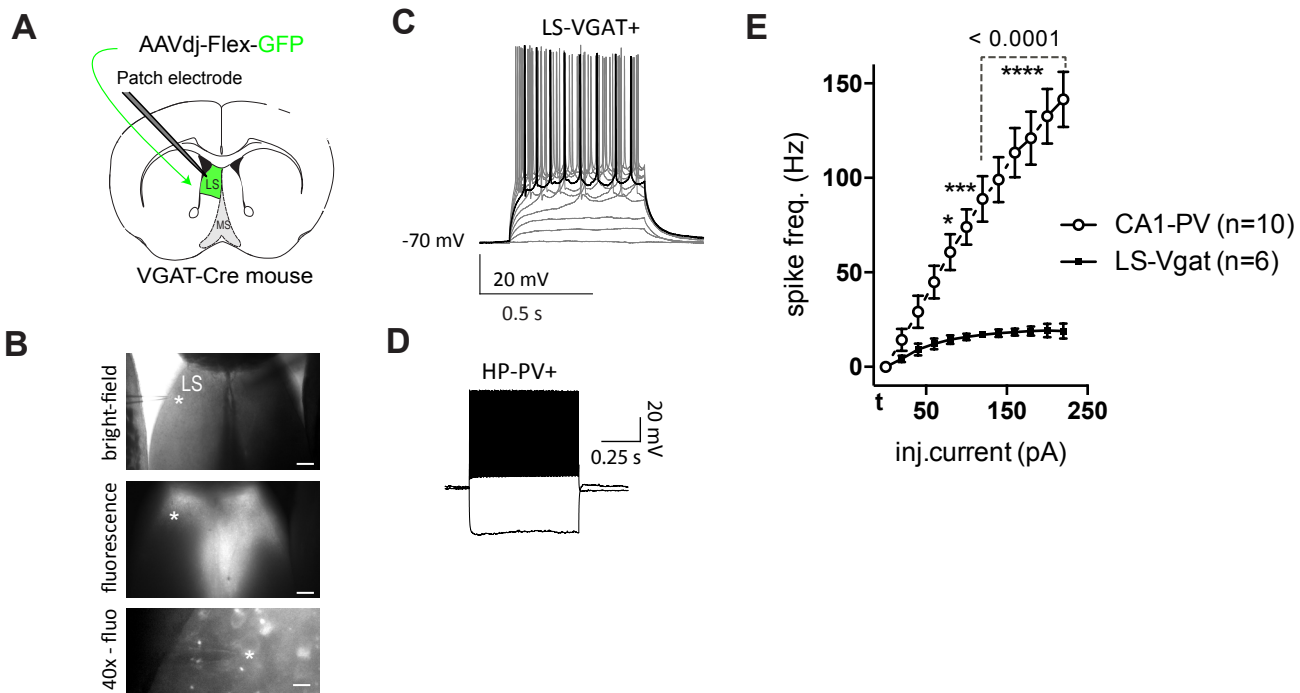

Supplement: S5 Fig — (A) VGAT-cre mice were injected with AAVdj-Flex-GFP in LS, and patch-clamp whole cell recordings were performed from fluorescent neurons of the dorsal LS. (B) Representative photos showing the localization of Cre-dependent fluorescence and VGAT-positive neurons 2 weeks after bilateral microinjections at LS coordinates. Scale bar: 200, 200, 10 μm, (asterisk marks the location of a patched cell). (C) Current-clamp traces from a GFP-positive LS neuron (shown in b) characterized using depolarizing current injection steps (0–200 pA). (D) Sample traces showing hyperpolarizing and depolarizing responses from a fast-spiking (PV) hippocampal interneuron. (E) Plot of mean firing frequencies in response to injected currents of increasing suprathreshold amplitudes in hippocampal PV interneurons (open circles) and LS VGAT neurons (solid squares). Current injections were 600 ms square pulses. Firing frequencies are plotted from threshold current (t) to t + 220 pA (two-way ANOVA with Bonferroni’s multiple comparison test, F(11,154) = 21.74, p < 0.0001 for interaction effect). *, p < 0.05, ***, p < 0.001, ****, p < 0.0001. The underlying data can be found in S2 Data. LS, lateral septum; PV, parvalbumin. (PDF) [file pbio.3001383.s007.pdf]

S6 Fig

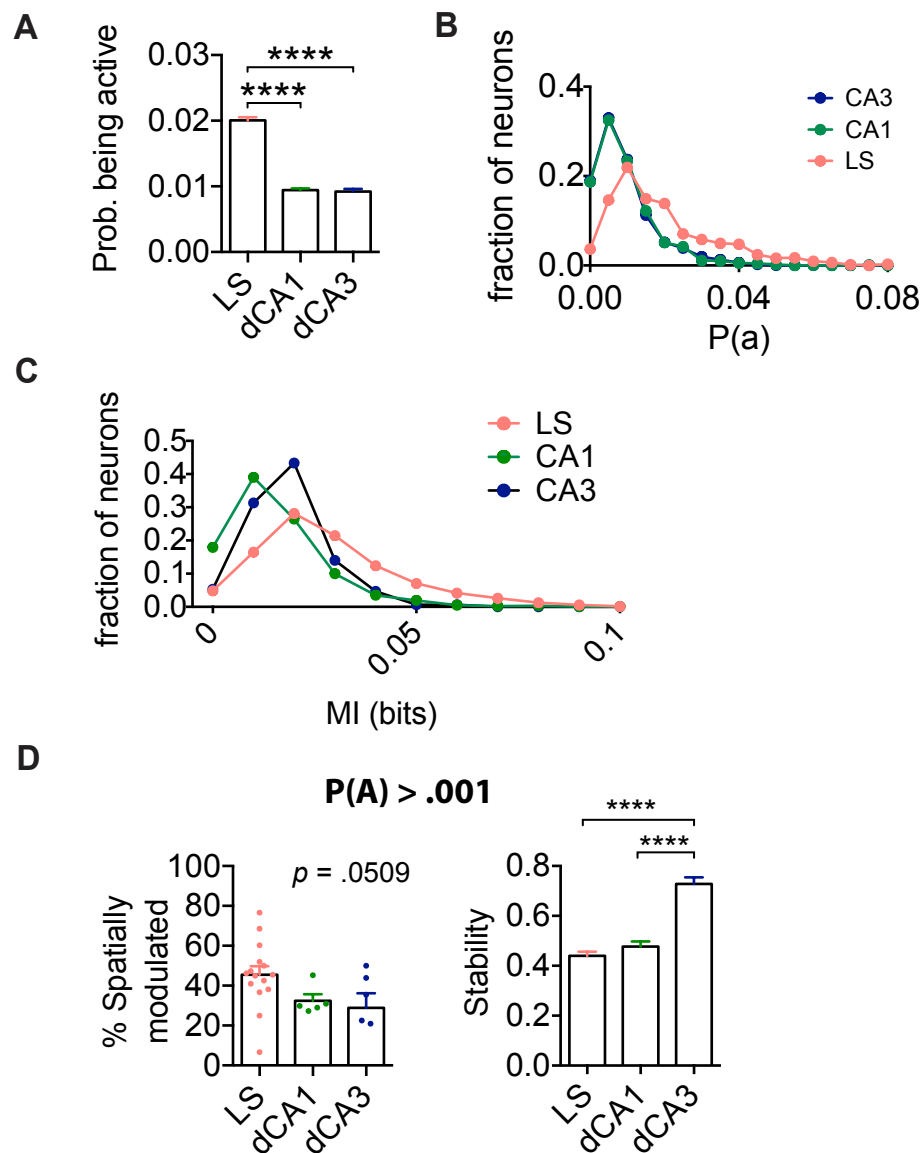

Supplement: S6 Fig — (A) Probability being active for all cells recorded for each group (Kruskal–Wallis, H(3) = 502.0, p < 0.0001; LS: n = 1,030 cells, n = 15 mice. CA1: n = 1,251 cells, n = 5 mice, CA3: n = 464 cells, n = 6 mice). (B) Histogram of probabilities being active for all groups (C) Histogram of MI values for all cells recorded in LS, CA1, and CA3. (D) Using a cutoff of P(A) > 0.001 to exclude cells of low activity levels, comparison of proportion of spatially modulated cells for each region (left, Kruskal–Wallis, H(3) = 59.57, p = 0.0509, LS: n = 15 mice. CA1: n = 5 mice, CA3: n = 6 mice) and average split half stability for spatially modulated cells (right, Kruskal–Wallis, H(3) = 80.48, p < 0.0001, LS: n = 475 cells, n = 15 mice, CA1: n = 363 cells, n = 5 mice, CA3: n = 142 cells, n = 5 mice). ****, p < 0.0001. The underlying data can be found in S2 Data. LS, lateral septum; MI, mutual information. (PDF) [file pbio.3001383.s008.pdf]

S7 Fig

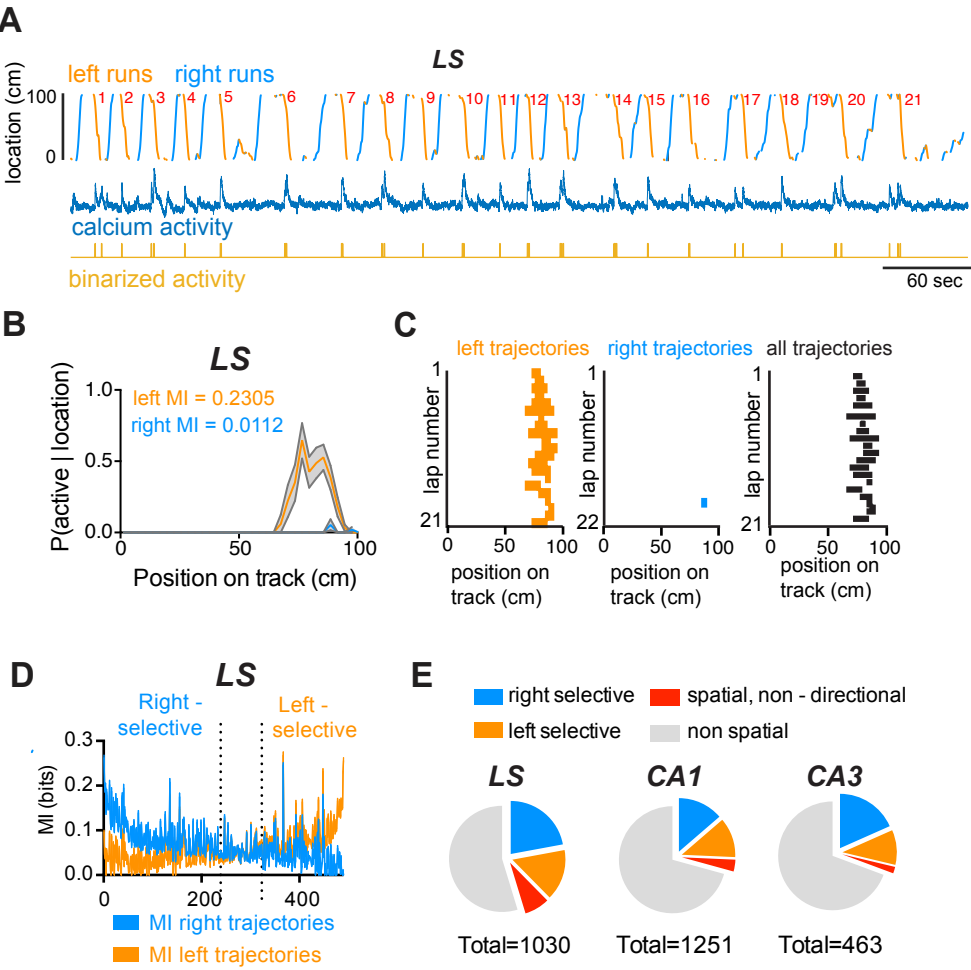

Supplement: S7 Fig — (A) Mouse location on day 5 of linear track training, divided in left runs (orange) and right runs (light blue) with corresponding raw calcium activity (middle, dark blue) and derived binary trace (bottom, yellow). (B) Probability of cell being active for left (orange) vs. right trajectories (blue), and corresponding MI calculated separately for left and right runs. (C) Corresponding locations where binarized activity was detected (orange for left trajectories, blue for right trajectories and black for all trajectories). (D) MI values for right vs. left trajectories ranked by magnitude of the difference between the two. Dotted line p < 0.01 significance level used to assess spatial modulation of cells, two-way ANOVA, F(489,28420) = 496.9, p < 0.0001 for interaction effect. (E) Proportion of spatially modulated cells in LS that are right selective (blue, 22.08%), left selective (orange, 15.52%), spatially modulated but not directionally modulated (red, 7.67%), and nonspatially modulated (gray, 54.71%, left), CA1 (middle) and CA3 (right). The underlying data can be found in S2 Data. LS, lateral septum; MI, mutual information. (PDF) [file pbio.3001383.s009.pdf]

S8 Fig

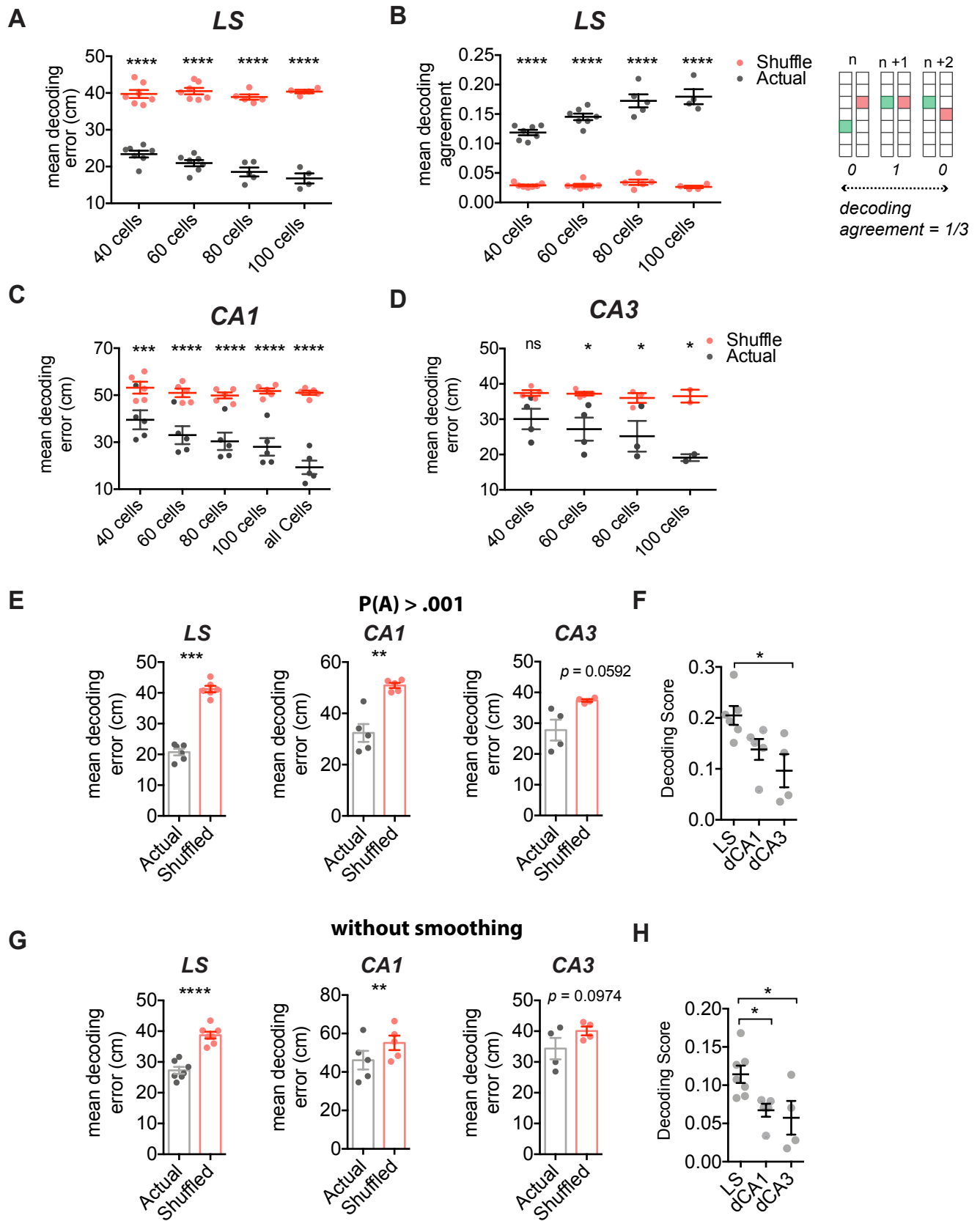

Supplement: S8 Fig — (A) Mean decoding error for location decoding on the linear track using LS cells computed for 30 bootstrap samples of 40, 60, 80, or 100 cells (black, each dot represents mean of an animal) compared to a decoded location using shuffled tuning maps (red, each dot represents mean of an animal), two-way RM ANOVA, F(3,19) = 571.4, p = 0.0024 for main effect of shuffling, F(3,19) = 6.948, p = 0.0024 for main effect of number of included cells. (B) Left: same as A, for mean decoding agreement, two-way RM ANOVA, F(3,19) = 1059, p < 0.0001 for main effect of shuffling, F(3,19) = 10.06, p = 0.0003 for main effect of number of included cells, Right: method for computing the mean decoding agreement for each bootstrapped estimate. (C) Same as A, for cells recorded from dorsal CA1. In addition to 30 bootstrapped samples of 40, 60, 80, and 100 cells, this panel includes mean decoding error using all recorded cells, two-way RM ANOVA, F(1,20) = 304.3, p < 0.0001 for main effect of shuffling, F(4,20) = 2.647, p = 0.0637 for main effect of number of included cells. (D) Same as A, for cells recorded from dorsal CA3, two-way RM ANOVA, F(1,9) = 42.83, p = 0.0001 for main effect of shuffling, F(3,9) = 1.480, p = 0.2845 for main effect of number of included cells. (E) Using only cells with P(A) > 0.001, decoding error for actual vs. shuffled dataset using 60 cells (paired t tests, LS: t(5) = 0.10, p = 0.001, n = 6 mice; CA1: t(4) = 6.732, p = 0.0025, n = 5 mice; CA3: t(3) = 2.966, p = 0.0592, n = 4 mice). (F) Using only cells with P(A) > 0.001, mean decoding score for each region (one-way ANOVA, F(2,12) = 5.765, p = 0.0167. (G) Same as E, without using a temporal smoothing window (paired t tests, LS: t(6) = 10.08, p < 0.0001, n = 7 mice; CA1: t(4) = 7.897, p = 0.0014, n = 5 mice; CA3: t(3) = 2.614, p = 0.0794, n = 4 mice). (H) Same as F, without using a temporal smoothing window (ANOVA, F(2,13) = 5.462, p = 0.0190). *, p < 0.05, **, p < 0.01, ***, p < 0.001, ****, p < 0.0001. The underlyi [file pbio.3001383.s010.pdf]

S9 Fig

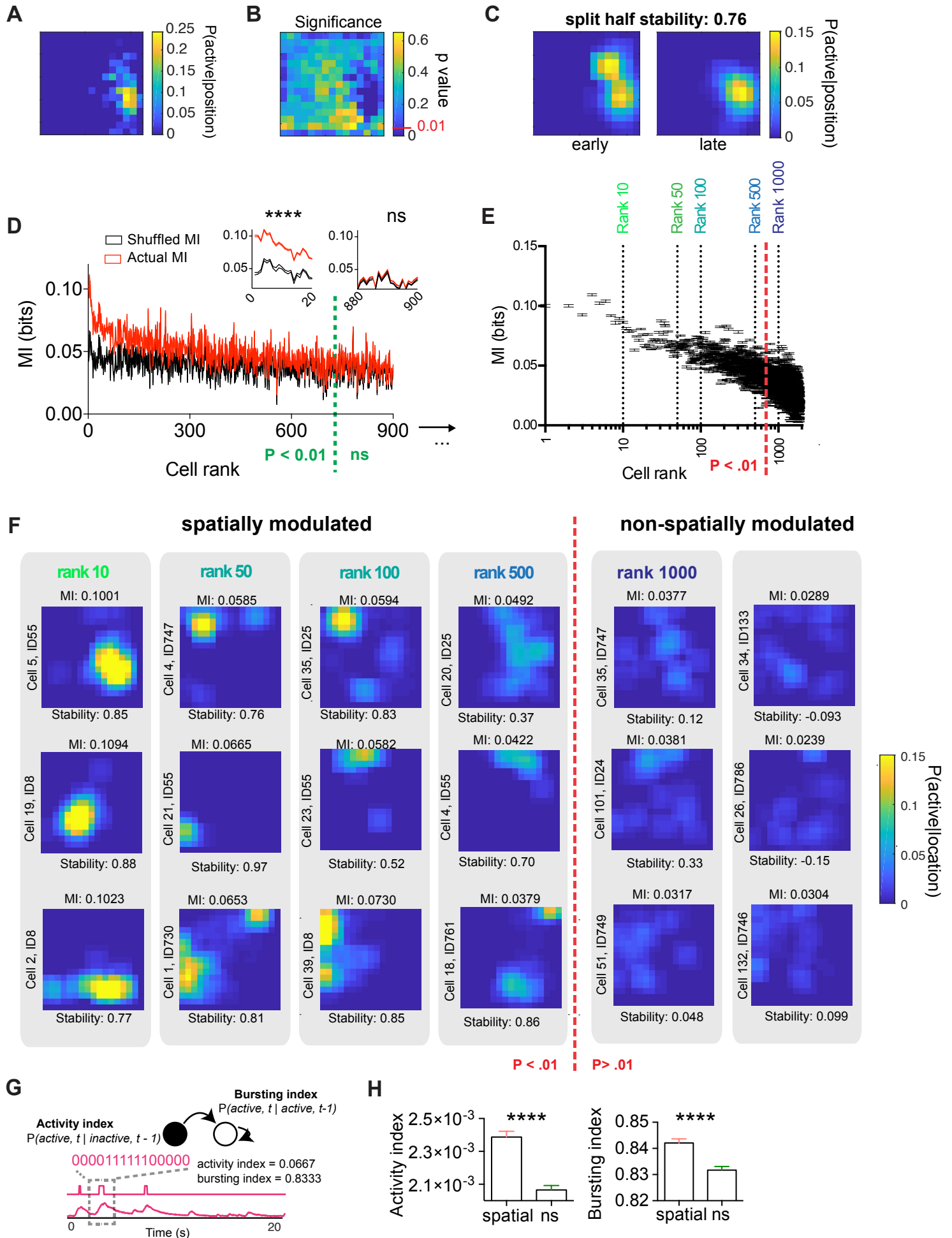

Supplement: S9 Fig — (A) Probability P(active | location) of an example cell to be active in a bin (3 cm) of the open field (45 or 49 cm size). (B) Significance computed from n = 1,000 circular permutations, p < 0.01 is considered significant. (C) Within-session stability is computed by correlation of the first half (left) and the second half (right) of the recording. (D) MI computed from actual traces (red) and shuffled traces (black), sorted by the magnitude of the difference between these values (one-way ANOVA, F(1898,110142) = 65.50, p < 0.0001 for interaction effect). Left inset: zoomed version of first 20 cells. Right inset: zoomed version for 20 not significant cells. (E) Cells are ranked according to the difference between their bootstrapped mean MI value and 30 circularly shuffled surrogates. Ranks are color coded for clarity, n = 1,899 cells from N = 28 mice. (F) Examples of significantly spatially modulated cells with corresponding mean MI value (top) and split within-session stability (bottom) representative for each rank. (G) Computation of activity index (probability inactive to active) and bursting index (probability active to active). (H) Left: activity index for spatial vs. nonspatial cells (Mann–Whitney test, U = 342,219, p < 0.0001). Right: bursting index for spatial vs. nonspatial cells (Mann–Whitney test, U = 368,276, p < 0.0001). The underlying data can be found in S2 Data. LS, lateral septum; MI, mutual information; ns, not signifiicant. (PDF) [file pbio.3001383.s011.pdf]

S10 Fig

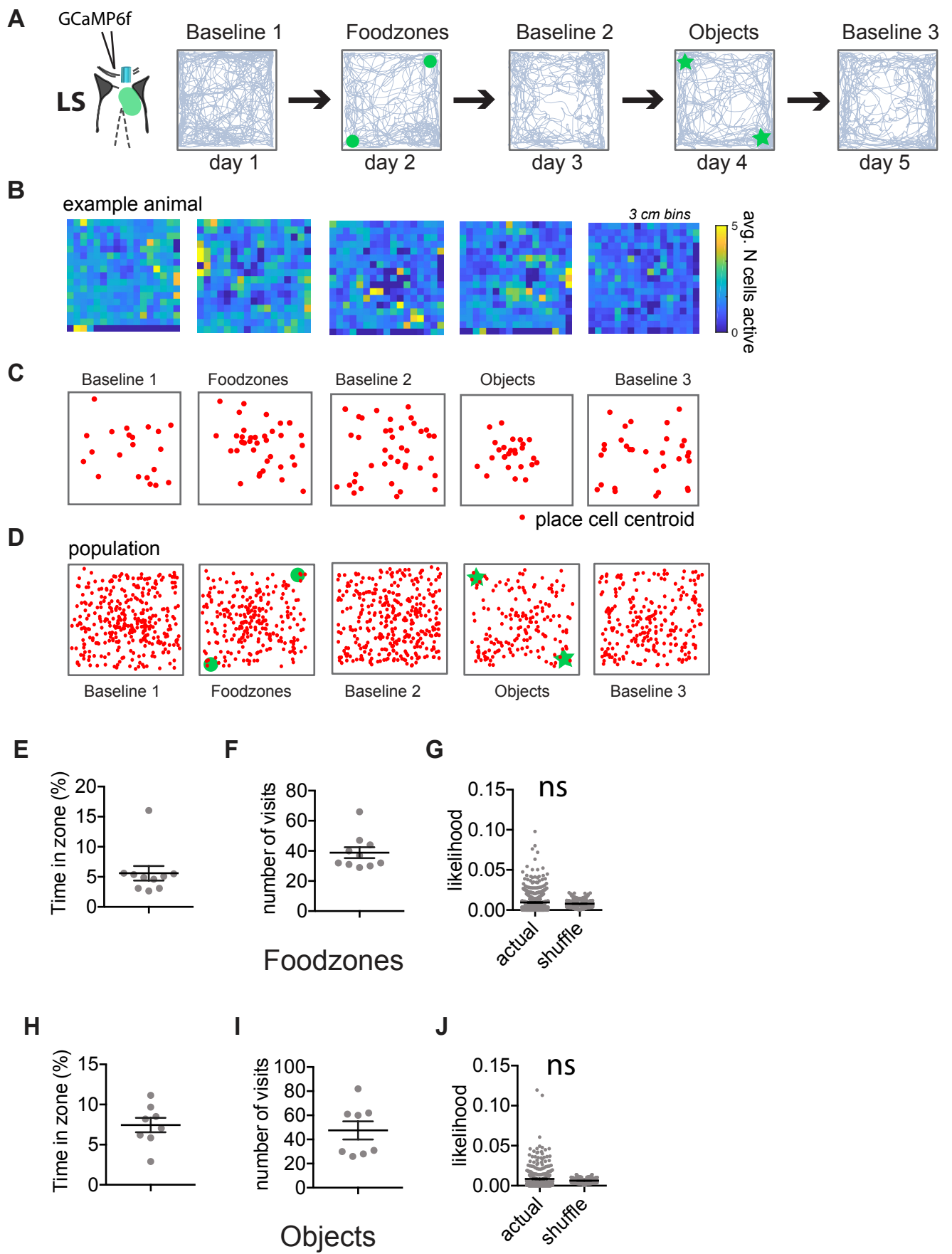

Supplement: S10 Fig — (A) Experimental setup, with animals freely exploring the same open field for 5 consecutive days, with 2 food zones and 2 objects added in opposing corners on days 2 and 4, respectively. Representative trajectories are shown in gray. Green circles and stars represent locations of food and objects. (B) Representative example of the average number of cells active per 3-cm bin for an example mouse (baseline 1: n = 132 cells; food zones: n = 110 cells; baseline 2: n = 118 cells; objects: n = 118 cells; baseline 3: n = 72 cells). (C) For the same animal, red dots are centroids of spatially modulated cells. (D) Centroids for each significantly spatially modulated cell of all animals included in analysis (baseline 1: n = 730 cells, n = 10 mice; food zones: n = 559 cells, n = 10 mice; baseline 2: n = 749 cells; n = 10 mice; objects: n = 407 cells, n = 8 mice; baseline 3: n = 471 cells; n = 8 mice). (E) Time spent in food zone as a percentage of total time spent in session for all animals (n = 10 mice). (F) Number of visits to food zone (n = 10 mice). (G) Likelihood of cell being active within the food zone as compared to a shuffle for all cells recorded (Wilcoxon matched-pairs signed rank test, W = −3,490, p = 0.6497, n = 559 cells, n = 10 mice). (H) Time spent in object zone as a percentage of total time spent in session for all animals (n = 8 mice). (I) Number of visits to objects (n = 8 mice). (J) Likelihood of a cell being active closely around the objects as compared to a shuffle (Wilcoxon matched-pairs signed rank test, W = 3,789, p = 0.4251, n = 407 cells, n = 8 mice). The underlying data can be found in S2 Data. LS, lateral septum; ns, not significant. (PDF) [file pbio.3001383.s012.pdf]

S11 Fig

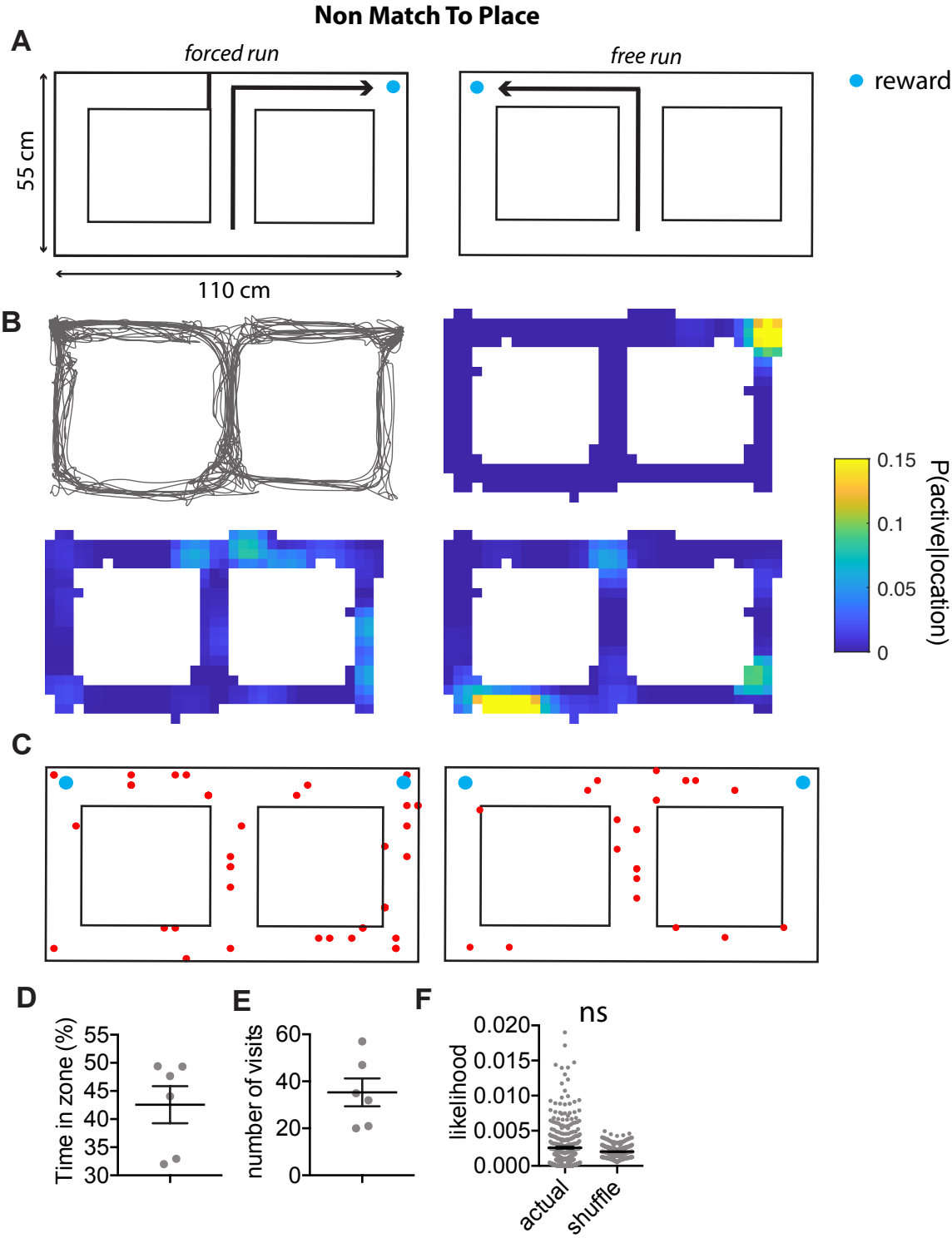

Supplement: S11 Fig — (A) Schematic of T-maze nonmatch to place task, consisting of a first forced run, followed by a free run in which the previously nonvisited arm is rewarded. (B) Example trajectory of well-trained animal, with Probability (active | location) of 3 example cells using 5-cm bins. (C) For 2 example animals, red dots are centroids of spatially modulated cells. (D) Time spent in food zone as a percentage of total time spent in session for all animals (n = 6 mice). (E) Number of visits to reward zone (n = 6 mice). (F) Likelihood of a cell being active within the reward zone as compared to a shuffle for all cells recorded (Wilcoxon matched pairs signed rank test, W = −4,783, p = 0.2358; n = 365 cells from n = 6 mice, day 10 of training). The underlying data can be found in S2 Data. ns, not significant. (PDF) [file pbio.3001383.s013.pdf]

S12 Fig

P(A) > .001

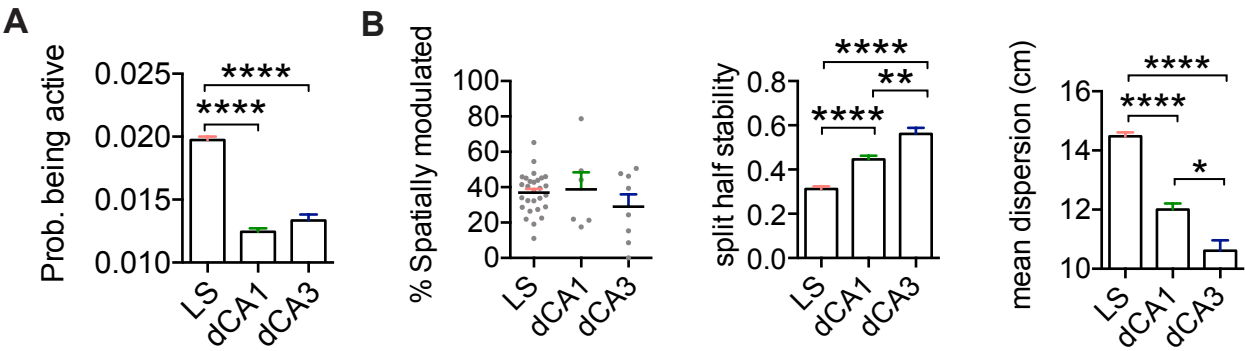

Supplement: S12 Fig — (A) Probability of being active for spatially modulated cells (Kruskal–Wallis, H(3) = 295.0, p < 0.0001; Dunn’s multiple comparisons test; LS: n = 718 spatial cells from n = 28 mice; dCA1: n = 323 spatial cells, n = 6 mice; dCA3: n = 138 spatial cells, n = 7 mice). (B) Left: using an activity cutoff of P(A) > 0.001, proportion of spatial cells per animal (one-way ANOVA, F(2,39) = 0.9849, p = 0.3826; Tukey’s multiple comparisons test; LS: n = 1,889 cells from n = 28 mice; dCA1: n = 1,017 cells, n = 6 mice; dCA3: n = 521 cells, n = 8 mice). Middle: within-session stability for spatial cells (Kruskal–Wallis, H(3) = 89.52, p < 0.0001; Dunn’s multiple comparisons test; LS: n = 718 spatial cells from n = 28 mice; dCA1: n = 323 spatial cells, n = 6 mice; dCA3: n = 138 spatial cells, n = 7 mice). Right: mean dispersion for spatial cells (Kruskal–Wallis, H(3) = 155.5, p < 0.0001; Dunn’s multiple comparisons test; LS: n = 718 spatial cells from n = 28 mice; dCA1: n = 323 spatial cells, n = 6 mice; dCA3: n = 138 spatial cells, n = 7 mice). *, p < 0.05, **, p < 0.01, ***, p < 0.001, ****, p < 0.0001. The underlying data can be found in S2 Data. LS, lateral septum. (PDF) [file pbio.3001383.s014.pdf]

S13 Fig

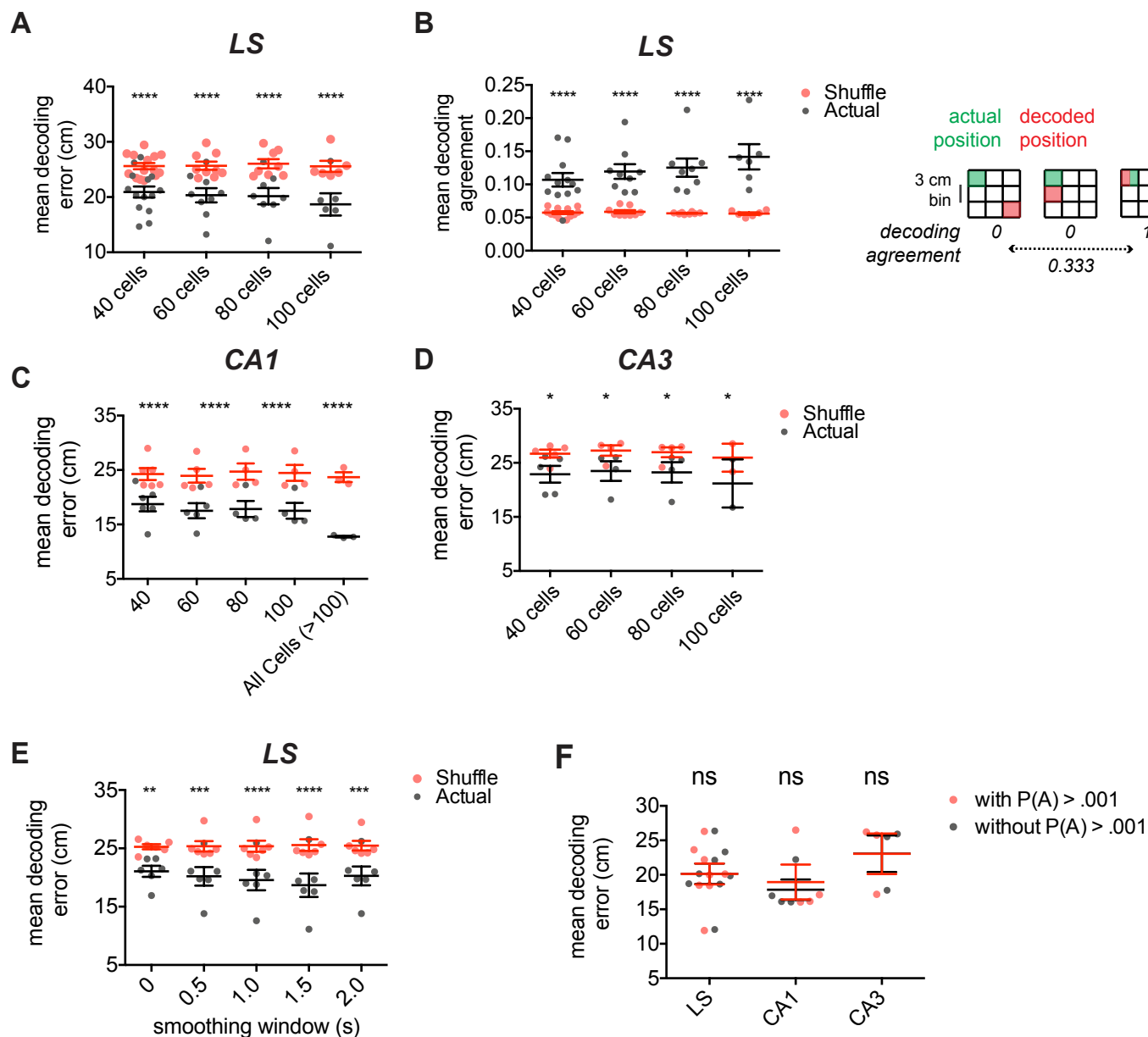

Supplement: S13 Fig — (A) Mean decoding error for location decoding in the open field using LS cells computed for 30 bootstrapped samples of 40, 60, 80, and 100 cells (black, each dot represents mean of an animal) compared to a decoded location using shuffled tuning maps (red, each dot represents mean of an animal; two-way RM ANOVA, F(1,33) = 174.9, p < 0.0001 for main effect of shuffling, F(3,33) = 0.2041, p = 0.893 for main effect of number of included cells. (B) Left: same as A, for mean decoding agreement (two-way RM ANOVA, F(1,33) = 132.8, p < 0.0001 for main effect of shuffling, F(3,33) = 1.132, p = 0.3505 for main effect of number of included cells). Right: method for computing the mean decoding agreement for each bootstrap estimate. (C) Same as A, for cells recorded from dorsal CA1. In addition to 30 bootstrap samples of 40, 60, 80, and 100 cells, panel includes mean decoding error using all recorded cells (two-way RM ANOVA, F(1,17) = 649.0, p < 0.0001 for main effect of shuffling, F(4,17) = 0.7948, p = 0.5447 for main effect of number of included cells) (D) Same as A, for cells recorded from dorsal CA3 (two-way RM ANOVA, F(1,11) = 44.13, p < 0.0001 for main effect of shuffling, F(3,11) = 17.99, p = 0.9078 for main effect of number of included cells). (E) Effect of temporal filtering on decoding error in the open field for LS (two-way RM ANOVA, F(1,25) = 123.7, p < 0.0001 for main effect of shuffling, F(4,25) = 0.1169, p = 0.9753 for main effect of number of included cells. (F) Comparison of mean decoding error using P(A) > 0.001 activity cutoff for cell selection vs. without such cutoff using 80 cells (LS: n = 8 mice, CA1: n = 4 mice, CA3: n = 4 mice; two-way RM ANOVA, F(1,12) = 1.374, p = 2.639 for main effect of activity cutoff, F(2,12) = 1.023, p = 0.3889, for main effect of structure). *, p < 0.05, **, p < 0.01, ***, p < 0.001, ****, p < 0.0001. Test used in A–F, two-way RM ANOVA. The underlying data can be found in S2 Data. LS, lateral septum; ns, not significant. (PDF) [file pbio.3001383.s015.pdf]

S14 Fig

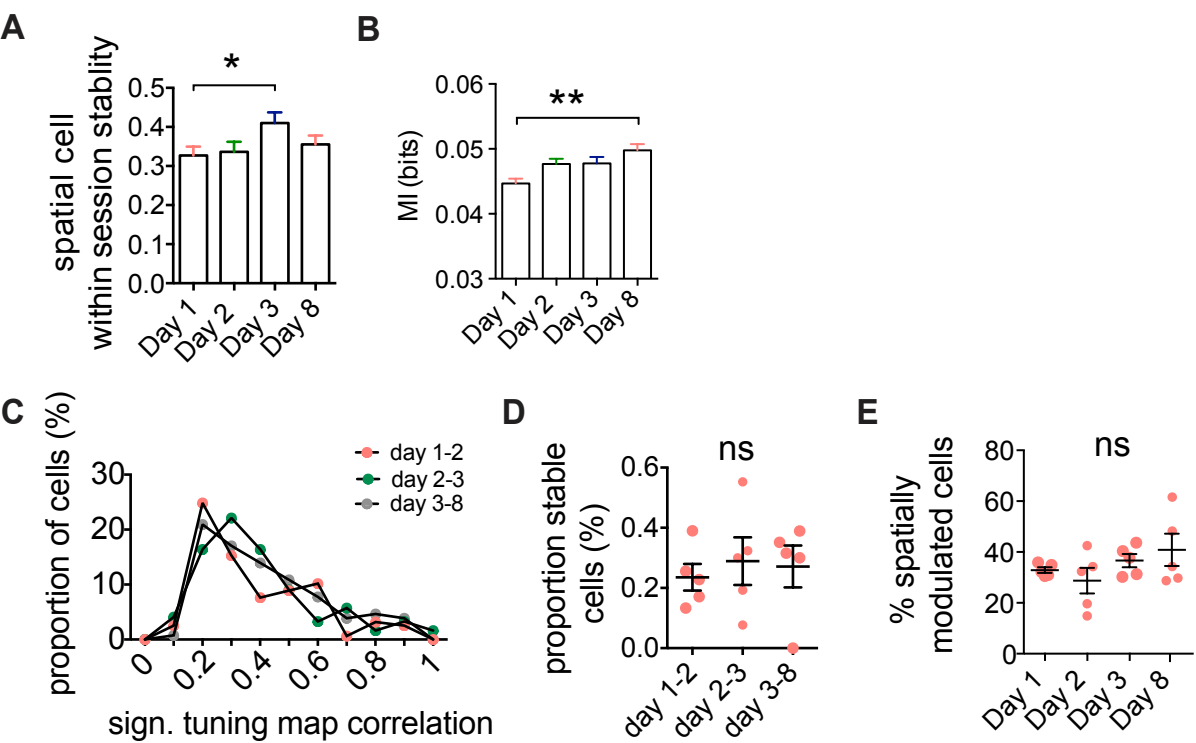

Supplement: S14 Fig — (A) Within-session stability for each spatially modulated cell recorded cells in LS (Kruskal–Wallis, H(4) = 8.921, p = 0.0304; Dunn’s multiple comparisons test; day 1, n = 181; day 2, n = 172; day 3, n = 178; day 8, n = 209, n = 5 mice). (B) Average MI for each day for all recorded cells (Kruskal–Wallis, H(4) = 13.27, p = 0.0041; Dunn’s multiple comparisons test; day 1, n = 562; day 2, n = 600; day 3, n = 477; day 8, n = 492, n = 5 mice). (C) Proportion of cells for tuning map correlation (Friedman test, χ2(2) = 0.400, p = 0.9537, significant cell pairs only, day 1–2, n = 158; day 2–3, n = 122, day 3–8, n = 129). (D) Proportion of stable cells (tuning map correlation > 0.3) for each progressive day correlation (one-way ANOVA, F(3,16) = 1.457, p = 0.2637; significant cell pairs only, day 1–2, n = 158; day 2–3, n = 122, day 3–8, n = 129). (E) Proportion of spatially modulated cells per day (day 1, n = 562; day 2, n = 600; day 3, n = 477; day 8, n = 492, n = 5 mice). *, p < 0.05, **, p < 0.01. The underlying data can be found in S2 Data. LS, lateral septum; MI, mutual information; ns, not significant. (PDF) [file pbio.3001383.s016.pdf]

S15 Fig

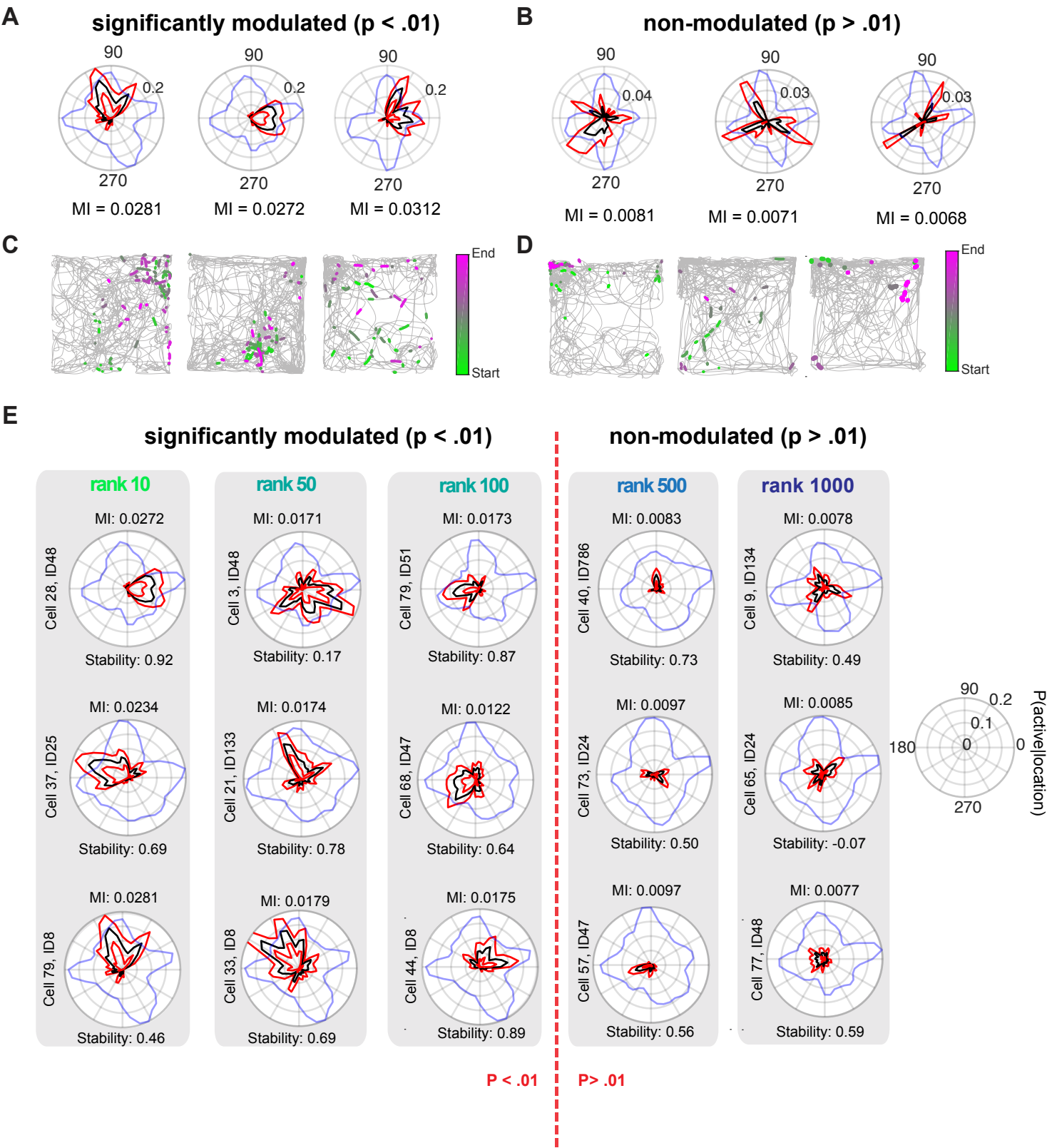

Supplement: S15 Fig — (A) Examples of significantly modulated cells. The polar plot indicates the probability of the cell being active as a function of the animal’s head direction. Black lines indicate p(active | bin); red lines indicate 95% upper and lower percentile; blue lines indicate the normalized time spent in each direction. MI calculated using 9° bins. (B) Same as A, for nonmodulated cells. (C) Trajectories (gray) with binarized activity superimposed, color coded from beginning to end of the recording for representative cells shown in A. (D) Same as C, but for example, cells shown in B. (E) Examples of significantly directionally modulated cells with corresponding mean MI value (top) and within-session stability (bottom) representative for each rank. The underlying data can be found in S2 Data. LS, lateral septum; MI, mutual information. (PDF) [file pbio.3001383.s017.pdf]

S16 Fig

A

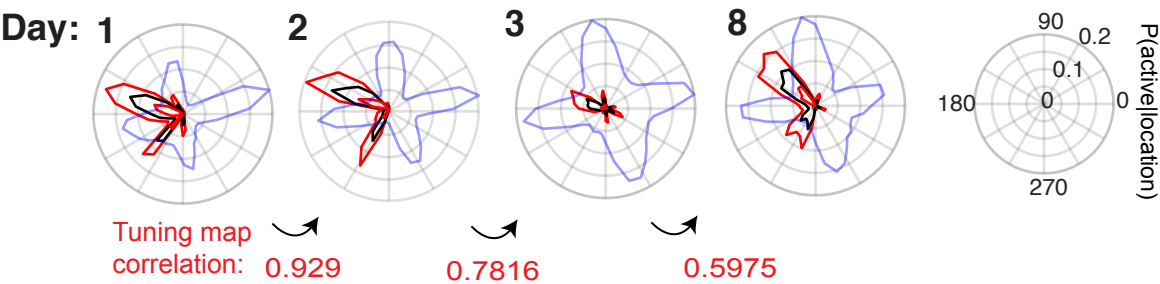

Direction

Direction

B

LS

CA1

C

LS

CA1

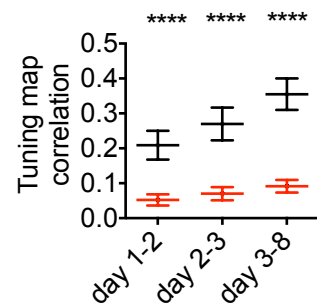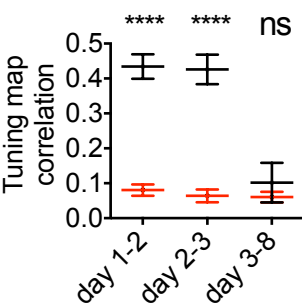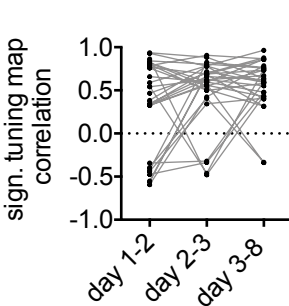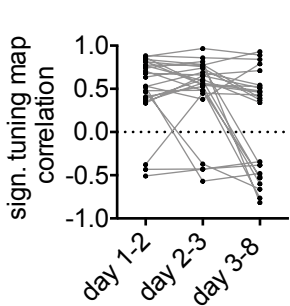

D

LS

CA1

E

LS

CA1

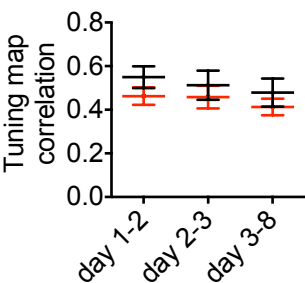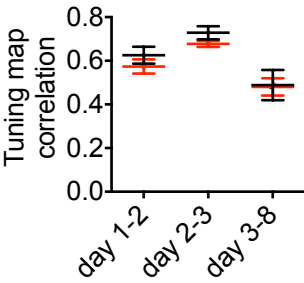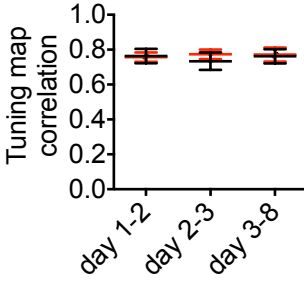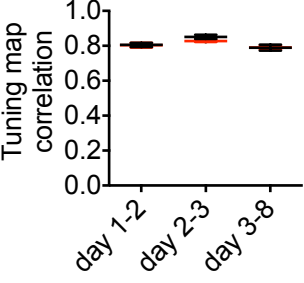

Supplement: S16 Fig — (A) Tuning plots for a stable directionally modulated cell over days, using a similar setup as described in Fig 4. Tuning map correlation indicated at the bottom in red. (B) Significant tuning map correlation for aligned cell pairs (black) vs. shuffled pairs (red) for progressive days for LS (day 1–2, n = 161 cells; day 2–3, n = 119 cells; day 3–8, n = 110 cells; n = 5 mice) and dorsal CA1 (day 1–2, n = 149 cells; day 2–3, n = 102 cells; day 3–8, n = 90 cells; n = 3 mice). (C) Significant direction map correlations for cells found on all days for LS (n = 29 cells) and CA1 (n = 24 cells). (D) Same as B, for velocity tuning in LS (day 1–2, n = 110 cells; day 2–3, n = 73 cells; day 3–8, n = 73 cells; n = 5 mice) and dorsal CA1 (day 1–2, n = 109 cells; day 2–3, n = 72 cells; day 3–8, n = 61 cells; n = 3 mice). (E) Same as B, for acceleration tuning in LS (day 1–2, n = 73 cells; day 2–3, n = 47 cells; day 3–8, n = 43 cells; n = 5 mice) and dorsal CA1 (day 1–2, n = 45 cells; day 2–3, n = 40 cells; day 3–8, n = 35 cells; n = 3 mice) ****, p < 0.0001. Test used in B, D, E: two-way ANOVA, with Sidak’s multiple comparisons test. The underlying data can be found in S2 Data. LS, lateral septum; ns, not significant. (PDF) [file pbio.3001383.s018.pdf]

S17 Fig

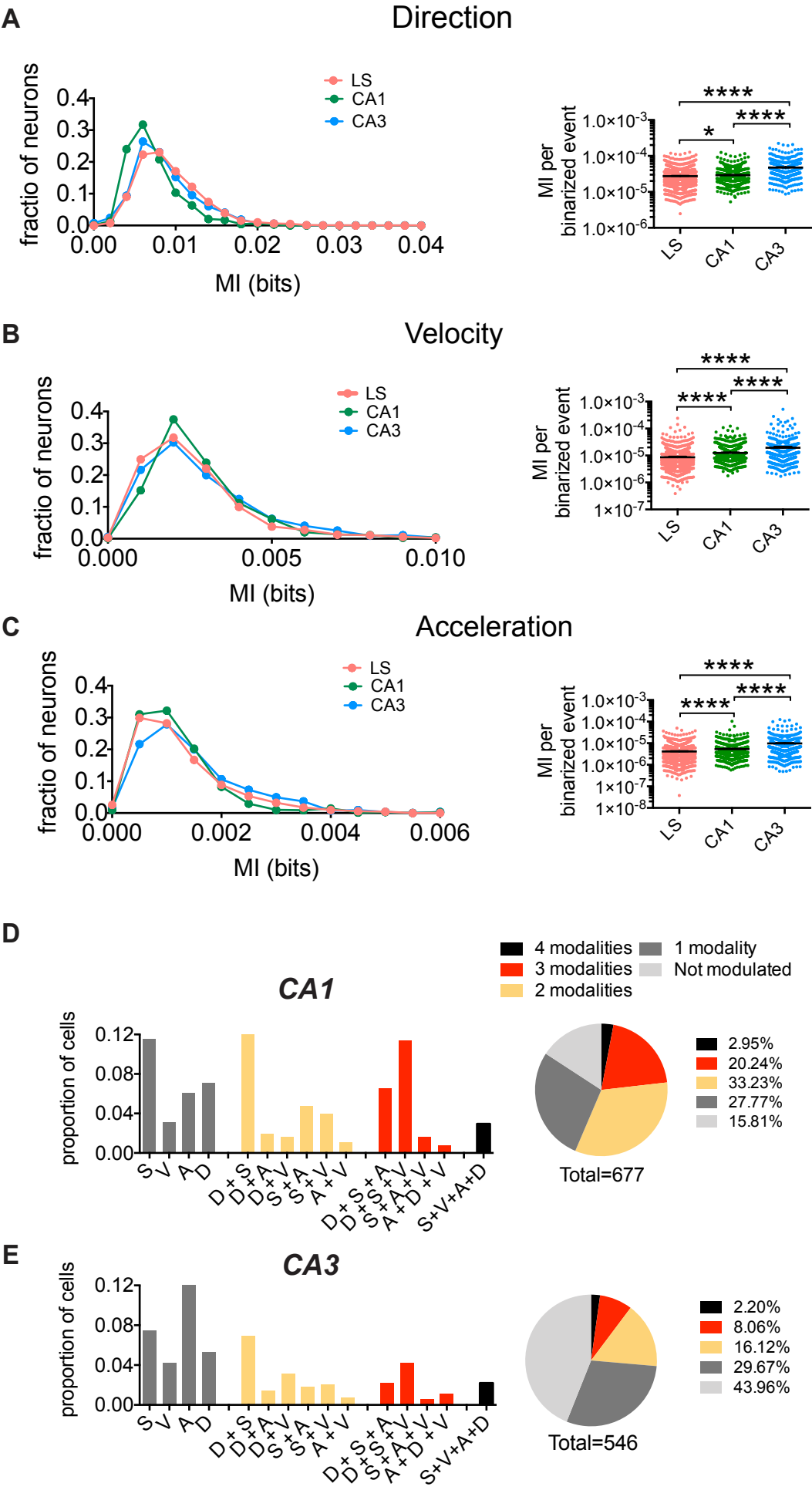

Supplement: S17 Fig — (A) Left: distribution of MI (left) and p-values (right) for direction for dCA1, dCA3, and LS. Right: MI (bits) per binarized event, for all cells recorded from each region (Kruskal–Wallis, H(3) = 351.2, p < 0.0001; Dunn’s multiple comparisons test; LS: n = 1,230 cells, n = 19 animals, CA1: n = 677 cells, n = 4 animals, CA3: n = 546 cells, n = 7 animals). (B) Same as A, for velocity (Kruskal–Wallis, H(3) = 325.3, p < 0.0001; Dunn’s multiple comparisons test) (C) Same as B, for acceleration (Kruskal–Wallis, H(3) = 262.2, p < 0.0001; Dunn’s multiple comparisons test). (D) Left: proportion of cells that are significantly modulated by only one modality (gray), 2 modalities (yellow), 3 (red) or all 4 of the investigated variables (black) for dCA1 (n = 677 cells, n = 4 mice). Right: absolute proportion of cells modulated by any combination of variables. E) Same as D, for dCA3 (n = 546 cells, N = 7 mice). *, p < 0.05, ****, p < 0.0001. The underlying data can be found in S2 Data. A, acceleration; D, direction; LS, lateral septum; MI, mutual information; S, spatial coding; V, velocity. (PDF) [file pbio.3001383.s019.pdf]

S18 Fig

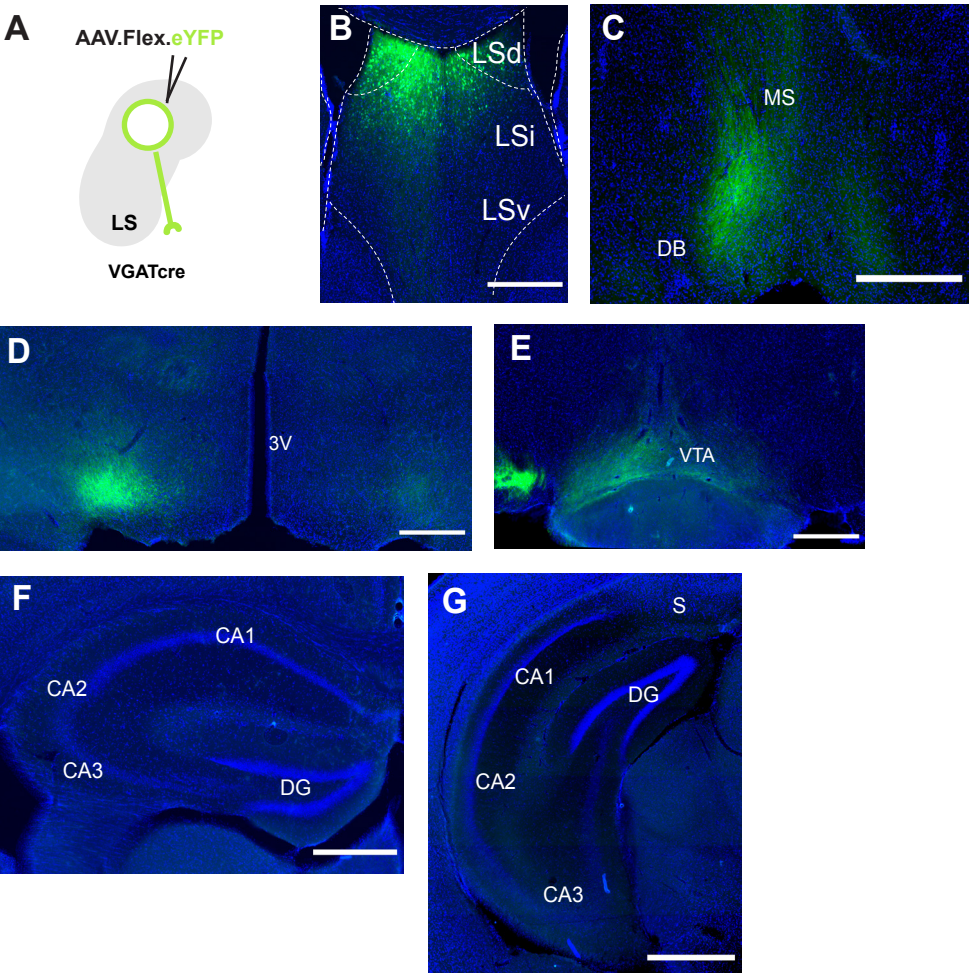

Supplement: S18 Fig — (A) Injection of anterograde, Cre-dependent eYFP (green) viral tracing in VGAT-Cre mouse LS. (B) Injection site in LSi/LSd with some cell bodies labeled in LSi (green, eYFP; blue, DAPI counterstaining). (C) eYFP-positive fibers in the MS, (D) hypothalamus, and (E) VTA. (F) Coronal hippocampal section shows no anterograde labeling of the hippocampal formation, either dorsal or (G) ventral. Scale bars: B, 500 μm; C, 500 μm; D, 500 μm; E, 500 μm; F, 500 μm; G, 700 μm. The underlying data can be found in S2 Data. DB, diagonal band; DG, dentate gyrus; LS, lateral septum; LSd, dorsal lateral septum; LSi, intermediate lateral septum; LSv, ventral lateral septum; MS, medial septum; S, subiculum; TA, ventral tegmental area; 3V, third ventricle. (PDF) [file pbio.3001383.s020.pdf]

S19 Fig

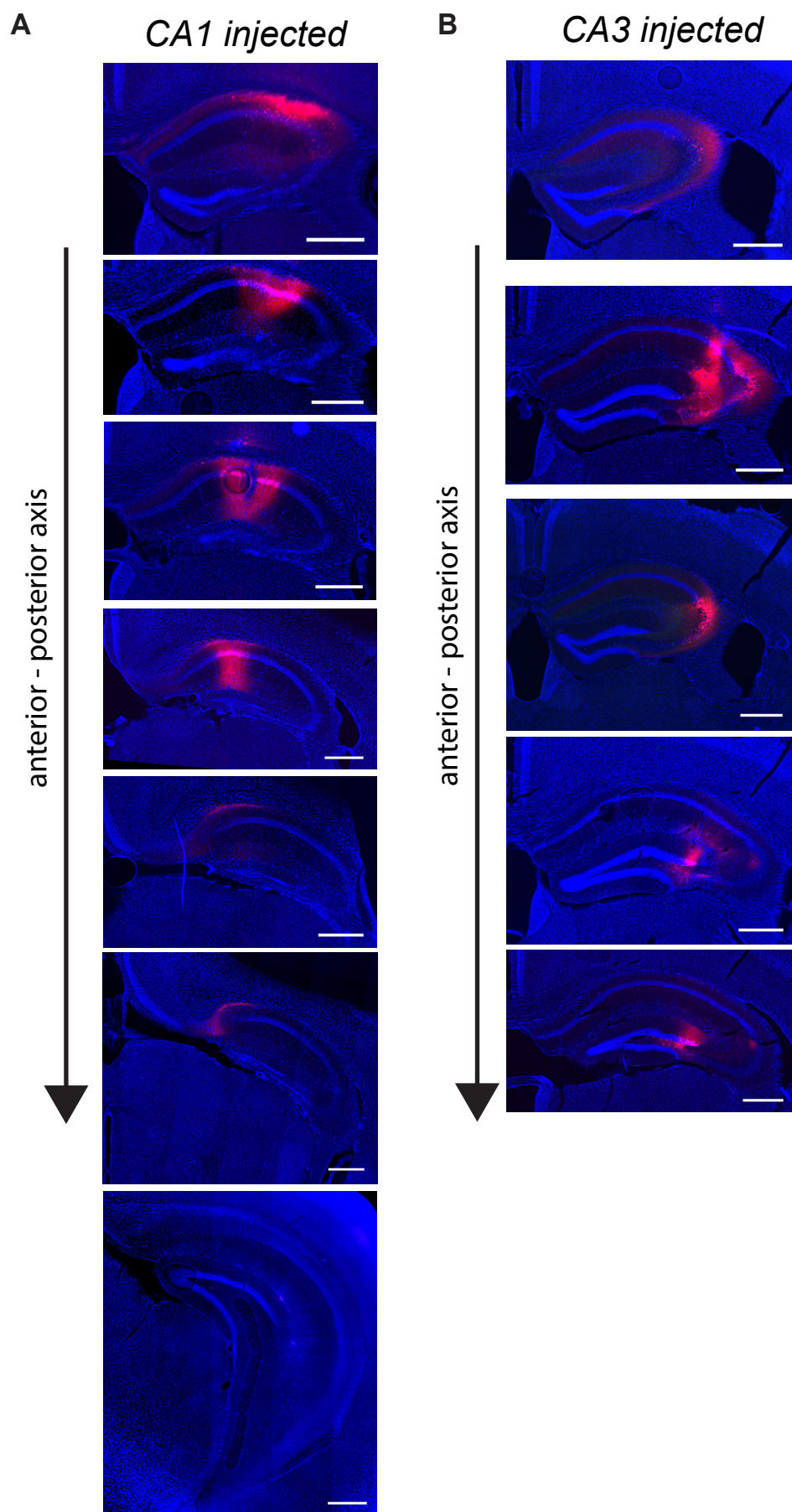

Supplement: S19 Fig — (A) Coronal sections showing expression of tdTom (red) in dorsal CA1 along anterior to posterior axis. (B) Same as A, for primary injections in dorsal CA3. Scale bars: 500 μm for all sections. The underlying data can be found in S2 Data. (PDF) [file pbio.3001383.s021.pdf]

S20 Fig

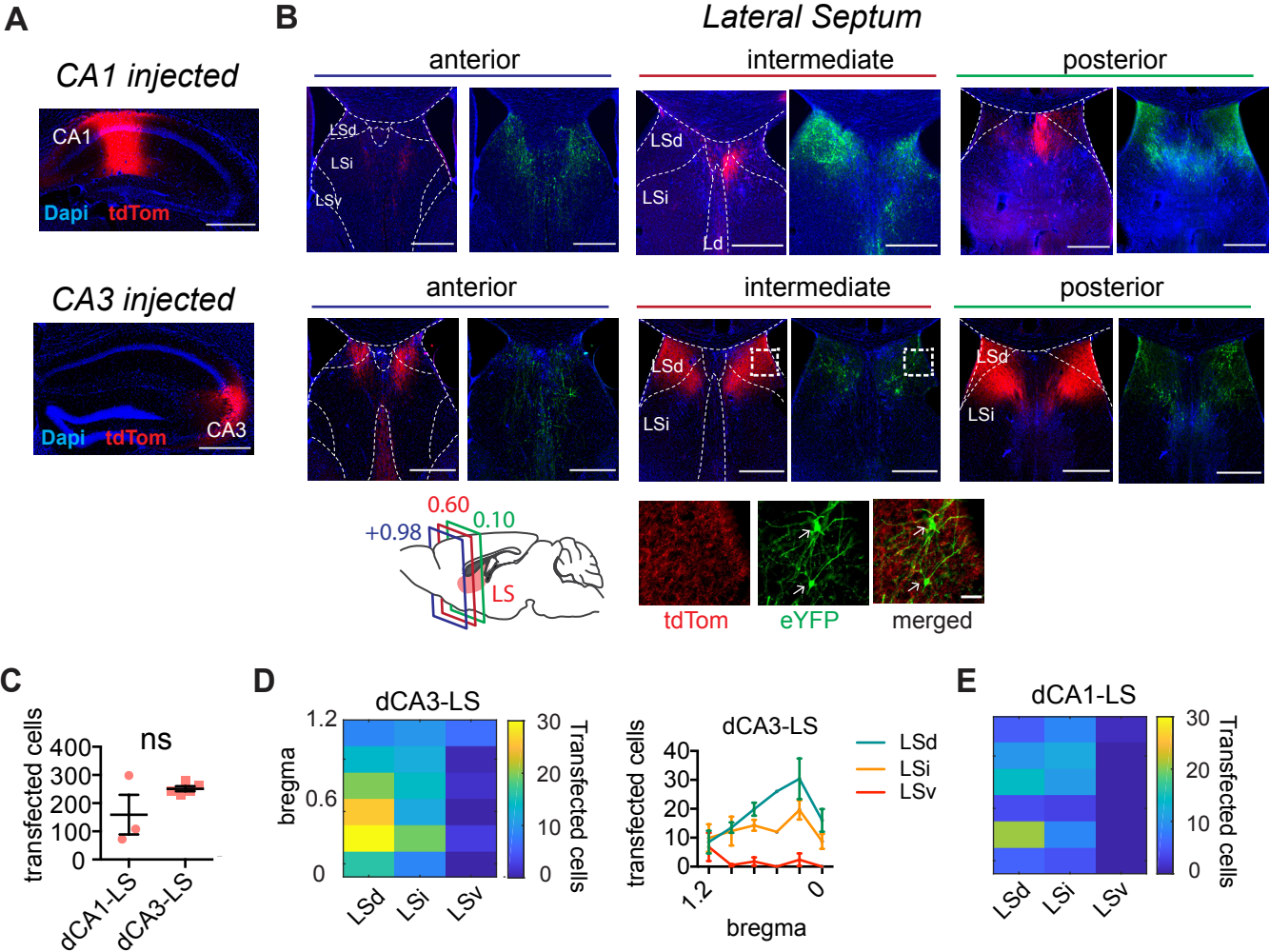

Supplement: S20 Fig — (A) Primary AAV1 injections in CA1 and CA3, using the same injection strategy as described in Fig 6A. (B) Coronal section showing expression pattern at different anterior–posterior levels of the LS, with (red) tdTOM-positive CA3 projections to LS and eYFP-positive second-order transduction in LS (left). Bottom schematic: an overview of the approximate bregma level of coronal slices shown. Bottom right: zoomed versions showing tdTom-positive hippocampal afferents, eYFP-positive LS cell bodies, and merge. (C) Total eYFP-positive cells counted at the level of LS following transsynaptic tracer injection in dCA1 vs. dCA3 (dCA1-LS, N = 3 mice; dCA3-LS, N = 5 mice). (D) Total eYFP-positive cells along the dorsal–ventral and anterior–posterior axes observed in LS for dorsal CA3 injection and (E) for dorsal CA1 injection. Scale bars: B, top: 500 μm; bottom: 500 μm. C, all overview images, 500 μm, all zoomed images, 50 μm. The underlying data can be found in S2 Data. LS, lateral septum; LSd, dorsal lateral septum; LSi, intermediate lateral septum; LSv, ventral lateral septum; ns, not significant. (PDF) [file pbio.3001383.s022.pdf]

S21 Fig

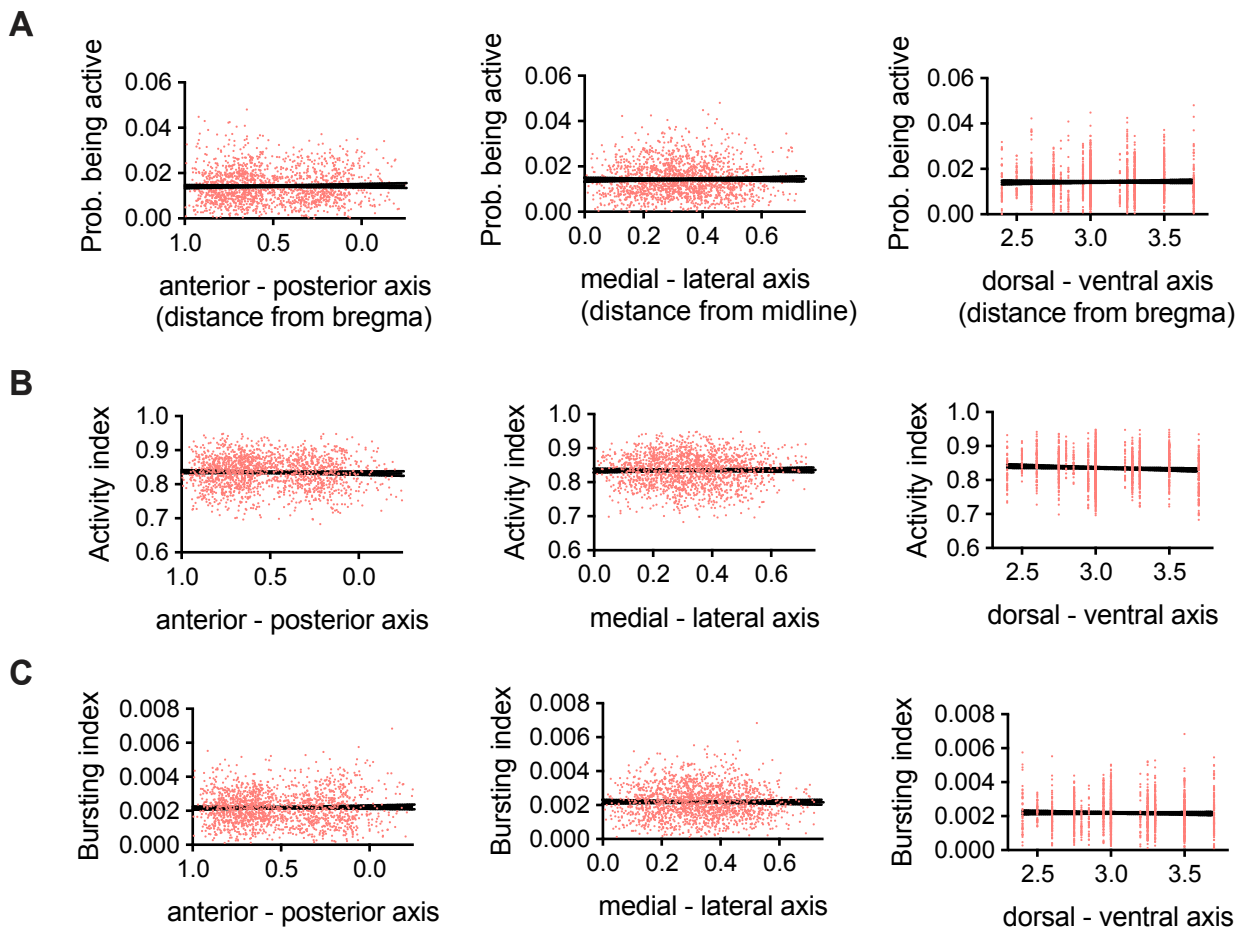

Supplement: S21 Fig — (A) Probability of being active for each cell along the anterior–posterior axis (left), medial–lateral axis (middle), and dorsal–ventral axis (right). All cells were recorded during free exploration in the open field (linear regression, AP: R2 = 2.014 × 10^4^, p = 0.5611; ML: R2 = 1.012 × 10^4^, p = 0.6803; DV: R2 = 2.717 × 10^4^, p = 0.6803; n = 1,679 cells, n = 24 mice). (B) Same as A, but for activity index as described in S5 Fig (linear regression, AP: R2 = 2.691 × 10^4^, p = 0.5017; ML: R2 = 3.287 × 10^5^, p = 0.8144; DV: R2 = 3.254 × 10^4^, p = 0.4601; n = 1,679 cells, n = 24 mice). (C) Same as for A, but for bursting index (linear regression, AP: R2 = 7.085 × 10^4^, p = 0.2757; ML: R2 = 7.457 × 10^5^, p = 0.7237; DV: R2 = 3.731 × 10^3^, p = 0.0123; n = 1,679 cells, n = 24 mice). The underlying data can be found in S2 Data. LS, lateral septum. (PDF) [file pbio.3001383.s023.pdf]
