# Supplementary material for: Conjunctive spatial and self-motion codes are topographically organized in the GABAergic cells of the lateral septum
Source: PLoS Biol. 2021 Aug 30;19(8):e3001383. doi: 10.1371/journal.pbio.3001383 (PMC8432898; doi:10.1371/journal.pbio.3001383)
Supplement: Attachment — Submitted filename: vanderVeldt_et_al_ResponseToReviewers.docx [file pbio.3001383.s024.docx]

Response to reviewers - Van der Veldt et al., 2021

-------------------------------------------------------------

Reviewer #1: Van der Veldt and colleagues imaged calcium transients in the lateral septum and hippocampus during spatial navigation. Thanks to their numerous recordings at different LS locations, they effectively investigate the topographical organization of the spatial and idiothetic codes within LS. The manuscript is well-written and provides by far the best account of the downstream copy of the hippocampal spatial code to LS. Overall, this is an excellent manuscript and I highly recommend it for publication. I have a few minor comments below.

*We thank the reviewer for taking the time to review our manuscript and positive comments.*

Line 141: we thus observed a significantly higher proportion of place cells in LS as compared to CA1 and CA3 (Kruskall-Wallis, H(3) = 6.102, p = .0298, Fig 1F).

Did the author group CA1 and CA3 for this comparison? It's unclear from the text or figure what is being compared.

*We did not group CA1 and CA3 for this comparison. We have updated this figure with more CA3 animals, and we have updated this section of the results. We rephrased this sentence in a way that better reflects the comparisons that were done (line 133 - 146).*

Line 143: We found that even though within-session stability of place cells was significantly different between LS, dCA1, and dCA3, stability in the LS was not significantly different than in dorsal CA1 (Kruskall-Wallis, H(3) = 8.922, p = .0116, Fig 1G).

Is LS different from CA3? The figure only indicates a difference between CA1 and CA3.

*We have now updated this figure and rephrased this section of the results to reflect that stability is only different across LS-CA3 and CA1-CA3, and not LS-CA1 (line 136-140).*

Line 204: In order to assess spatial coding during free exploration in a two-dimensional environment, we recorded Ca2+

Superscript for 2+ is missing.

*We have now corrected this in the new version of the manuscript (line 218)*

Line 210: we did not observe any increased number of place cells with fields around food zones, nor did we observe overrepresentation of objects or walls.

Provide a quantification of the time spent eating and exploring the objects. A simple explanation for not seeing any modulation around the food zones and objects could be that the mice failed to eat or explore. In addition, did you look at the calcium event rate in the rewarding zones?

*We are now providing a quantification of the % time spent and number of visits to the region of interest (food zones and object zones). This has been added to S10 Fig. We also compared the likelihood of activity in those ROIs and found no significant difference compared to shuffled surrogates, for both food and objects.*

Line 349: We observed that dCA1-LS preferentially targets the LH, and dCA3-LS targets the hypothalamus more broadly as well as the nuclei of the medial zone.

Perhaps you could compare this result to the proposed topographical projection from hippocampus to hypothalamus (Risold and Swanson, Science 1996)

*We have now added this in the discussion, line 577-580. We have also added this citation in the results section line 384-385 as we felt it would be appropriate there too.*

I couldn't find where Supplementary figure 9 is described in the main text.

*We apologize for this omission. The original Supplementary figure 9 is now referred to as S18 Fig, and we describe this figure in lines 383-386.*

Line 358: functional projections to medial septum neurons.

I think the word "functional" does not apply here and should be removed since the data presented is solely neuronal tracing.

*We agree with the point raised by the reviewer and have now rephrased this sentence, and no longer use the word functional to describe the projections from the LS to the MS (line 403).*

Line 966: (A) Dual-viral injection strategy same as described for S10 Fig.

Do you mean Fig. 5A? In addition, the identical color code (red, blue and green) used for the bregma coordinates (C) and the dorso-ventral localization in LS (F) is slightly confusing.

*We are now referring to the correct figure in line 1222-1223. We agree with the reviewer and we have changed the color code used in panel F (S20 Fig) to avoid any confusion.*

Line 385: Both dorsal dCA1 and dCA3 injections resulted in the strongest transfection of LS cell bodies in dorsal regions compared to intermediate and ventral LS (S10D-F Fig).

I agree with the conclusion but since the proportion of transfected cells will depend on where the Cre-dependent virus is injected (AP: 0.5; ML: 0.35; DV: -2.6 = dLS), I would prefer if you mentioned this possible caveat. Overall, if you want to conclude on the density of hippocampal innervation to LS, the pattern of TdTomato-labelled fibers is probably more indicative.

*We have mentioned this possible caveat in line 430-433. We rephrased our the conclusion of this experiment to better reflect the experiment, and changed it to ‘Hence, LS neurons receiving direct dorsal hippocampal input appear to be localized at more dorsal-posterior regions as compared to more ventral-anterior regions of LS’, line 434-437.*

Fig 6C: typo in the y-axis labels: "Proporton"

*We have now changed the naming of the y-axis labels.*

Line 609: injected in the LS of CaMKIIα-Cre mice.

Line 353: We used an AAV-Flex-Synaptag...

Why not inject a non-Cre-dependent synaptag virus in WT mice or the FLEX synaptag following trans-synaptic Cre-delivery from hippocampus to LS? Ad in the main text that the virus was injected in a CaMKII-Cre mouse. In this and a few other occasions it's a little unclear in what region the viruses were injected.

*We agree with the reviewer on these points. We used a CaMKII-cre mice as this marker is found at a much higher density in the LS compared to the MS. Using this marker prevented any leakage of viral expression in the MS, and thus ensured that we specifically targeted the LS to MS projection (now added in line 399-402). We have now also added the exact coordinates of these injections in our methods section (line 658-659).*

*We agree that the approach of injecting a transsynaptic AAV1 virus in CA1, followed by a Cre dependent Synaptag virus would be of interest, as it would provide further insights into whether those LS cells receiving direct inputs from the hippocampus project directly to the MS.*

Line 497: The question arises of what the function of could be having a region downstream

Correct the sentence.

*We apologize for the typo and have now corrected this sentence (now line 547)*

----------------------

Reviewer #2: In this manuscript, van der Veldt and colleagues recorded the activity of GABAgic neurons in the LS using in vivo calcium imaging technique. The authors report that the activity of the cells is modulated by place, speed, acceleration, and direction. The information content of these cells is comparable with those of hippocampal cells. The distribution of cells showing these properties formed gradients along A-P and D-V axes of the LS. Long-term tracking of LS neuronal activity revealed that a subset of LS cells has stable spatial representation over days. Using viral tracing they showed that LS neurons receive CA3 and CA1 excitatory input and send projections to the hypothalamus and medial septum.

Overall, this study presents multiple new findings, and the manuscript is well-written. However, interpretation of spatial distribution of place/kinematic cells in the LS seems somewhat difficult, because the invasiveness of endoscopic imaging. In my opinion, the manuscript is still suitable for publication in PLOS Biology, after the authors have addressed the following comments and questions.

1. Spatial organization of information in the LS (Fig. 6) is potentially very interesting, however, insertion of the GRIN lens significantly damages the local circuits when imaging from ventral part of the LS. Because the LS GABAgic cells are highly interconnected, imaging with GRIN lens would perturb their activity. In order to confirm the findings, LS activity should also be measured electrophyisiologically (e.g. tetrode recording).

*We agree with the reviewer that it could be of interest to confirm these findings electrophysiologically, however, gathering such a large dataset using tetrodes was unfortunately impossible for us within the time window of this review. We agree that a future study looking at this would be interesting.*

2. The authors reported that, contrary to the previous report, the LS place cells did not show overrepresentation of reward zone (Fig. S6). This discrepancy is likely due to the task demand: According to a previous report (Dupret et al., 2010 Nat Neurosci, 8 995), overrepresented activity around reward zone in the CA1 was observed only when the reward was hidden and the animals should navigate themselves to the reward zone using their spatial memory. Therefore, the following points should be clarified:

1. Was food or foodzone visible for the animals?

2. Did the CA1 cells show overrepresentation of reward in the same experimental condition?

*In our experiments, the foodzone was visible, which is now described better in the methods section (line 709-714). We did not record CA1 in these conditions. In our revised manuscript, we have now expanded our analyses of % of time spent in zone as well as number of visits to zone (S10E-F,H-I, Fig). Additionally, we are now showing the cell likelihood of being active in food zones compared to shuffled surrogates (S10G,J Fig). We also added LS recordings of mice (n = 6) performing a delayed non-matching to place task in a T-maze to assess reward-modulation in the context of increased task demand, but did not observe any significant reward code compared to shuffled surrogates (S11 Fig, line 244-247).*

3. Fig. 3B: The authors found that some LS cells show highly stable place representation. What percentage of the cells shows such a high stability? It would be more informative if graph showing tuning map correlation vs cell number is added.

*We have expanded on the analyses for this figure. In addition to mean tuning map correlation (Fig 4C), we are now also showing tuning map correlation for individual place cells (Fig 4D). In addition, we have added a histogram of significant positive tuning map correlations versus proportion of cells across all day pairs (S14C Fig).*

4. In addition to spatial representation, is self-motion information represented stably in LS cells?

*We are now showing the stability of self-motion information over days in S16 Fig (line 336- 343). We are showing mean tuning map correlation for direction (S16B Fig), velocity (S16D Fig) and acceleration (S16E Fig), and observed a significant stability over days for direction tuning compared to shuffled surrogates. We also assessed the tuning correlations for individual cells found on all 4 recording moments in S16C Fig.*

5. Fig. 3G: It seems slightly higher correlation for shorter interval in CA1 than in LS. Tuning map correlation for 1-day-interval as well as for day 1-8 should be quantified.

*We have now updated this graph and added the requested comparisons for all intervals in Fig 4F.*

6. Page 20 line 497: The authors discuss the possibility that the LS cells use sparse code. However, I could not find any data showing that activity of LS cells is more sparse than that of hippocampal cells. As shown in Fig. 2D, LS cells are more active than CA1/CA3 cells, and Fig. 2E showed that place field size is larger in LS than in CA1/CA3. These data are opposed to sparse coding.

*We apologize for the inappropriate terminology used in the original version, and understand that sparse coding implies lower activity rates (whereas LS cells are typically more active than CA1/CA3 cells). Instead, LS codes are more distributed (increased area coverage). We have now corrected the manuscript, discussion section, around line 561-563.*

Minor Comments:

1. Fig. 3A: GCAMPF6f -> GCAMP6f

*We have now corrected this error (Fig 4A). We thank the reviewer for the time and care put into reviewing our manuscript.*

Reviewer #3: Summary:

VanderVeldt et al present here a very interesting dataset of 1000+ fast-spiking neurons recorded with miniscopes covering a large extent of the lateral septum (LS) of mice freely behaving on a linear track or in an open field. They analyze how the activity of these neurons is modulated by spatial, directional and self-motion correlates and compare their findings with another dataset they recorded in similar conditions from the CA1 and CA3 regions of the hippocampus. Spatial, directional and self-motion coding had already been reported multiple times in the LS. Yet, the reported proportions of LS neurons modulated by these correlates vary a lot from on study to the other. VanderVeldt et al hypothesize that these discrepancies stem from an anatomical topographic organization of spatial coding. This hypothesis had already partially been explored by previous studies which did not find neither medio-lateral (Wirtshafet and Wilson 2020) nor dorso-ventral (e.g., Takamura et al., 2006) organization, yet they did not have access to a dataset as large as the one presented here.

I agree that that the use of miniscopes is indeed well appropriate to thoroughly test the existence of a topographic organization of spatial coding in LS, as well as to reconciliate apparent discrepancies within the literature. However, the results on topography only come in figure 6 and most of the findings presented prior to figure 6 are not very novel. Most of the data presented here aims to compare coding in LS fast-spiking GABAergic cells and CA1 pyramidal cells. I have several concerns related to the methods used by the authors to compare these very different populations that I will outline below. Moreover, the novelty of these findings is not striking, given that Wirtshafet and Wilson had already done so in a study published in elife in 2020.

In addition to their imaging studies, VanderVeldt et al present results from tracing studies to demonstrate a topographical organization of CA1-to-LS projections that they claim to correlate with the coding organization. Their main conclusion being that LS is a relay between the hippocampus and other subcortical structures such as the medial septum (MS) and the hypothalamus. I am not sure how much new results is provided here compared to previous studies. Moreover, the method here are not always appropriate to reach the conclusions outlined in the main text - see detailed concerns below. This is especially problematic when it comes to their main conclusion on LS being a relay. This conclusion cannot be reached based on the results presented here. It could be tested with targeted inactivation studies, which would be a whole other set of experiments.

Recommendation:

I agree that the question of coding in LS neurons (and its topographical functional organization) is very important to understand the neural mechanism supporting information processing, especially with regards to the putative role of LS as a hippocampal relay. I appreciate the interesting and rich dataset provided here, as well as the attempt at using tracing studies to supplemental their claims based on the miniscope results. However, I am not sure whether the findings of VanderVeldt offer- as for now - a substantive increase in our knowledge on that subject (except for figure 6). The promise of "functionally characterize hippocampal connectivity of LS" is very appealing but I do not think that the work presented here fulfill it. I was also put off by many imprecisions both in the text and in the figures, with incomplete legends or even a supplementary figure 9 that is not referred to within the text. My opinion is that this work will need major rewrite/rework/refocus in order to reach the quality and novelty of findings expected from articles published in PlosBiology. I am listing below my principal major and minor concerns, yet, given the high number of imprecisions, this is not an exhaustive list.

Major concern:

-	As stated in the summary above, my main concern is on the lack of novelty of the results presented here. The main advantage of VanderVeldt et al dataset over previous studies is the use of miniscope which allows to image simultaneously a large number of neurons, depict their precise anatomical location and morphology, as well as their activity in freely behaving animals, over several consecutive days. I don't think that this rich dataset was exploited to its fullest here, especially when reporting/comparing results with CA1 imaging. I urge the authors to highlight better, especially in their results portions, how what they present here is new and contrast with what has already been published.

*We thank reviewer #3 for exhaustive review and very helpful comments. We acknowledge that the novelty of our study should be further contrasted in the manuscript. We hope that we strengthened the new version of our manuscript by including the reviewers analysis and rephrasing suggestions. As the reviewer points out, (1) our study centers around a rich dataset acquired by in vivo calcium imaging in freely behaving mice, which we combined with an unbiased information theory approach and Bayesian decoding techniques (discussed in line 486-489). Moreover, (2) our study provides a first assessment of LS spatial coding over longer timeframes (up to 8 days), and strikingly, we observe a proportion of LS place cells that are stable across days, similarly to CA1 place cells. (discussed around line 496) (3) We find that directional information, acceleration, and velocity are conjunctively encoded in LS cells. To our knowledge, this constitutes the first description of such conjunctive coding in the LS (line 528 -533) (4) We performed a thorough and comprehensive characterization of the anatomical localization of spatial, head direction, and velocity cells in the LS structure (line 532-539).This work addresses the highly variable estimates of place cells in the LS, ranging from almost absent (Tingley & Buzsáki, 2018) to relatively few (Takamura et al., 2006) to abundant but of inferior quality (Wirtshafter & Wilson, 2019). (5) Finally, our recording and tracing results using trans-synaptic and monosynaptic viral techniques suggest that the LS accurately integrates information from the hippocampus and provides spatial, directional and self-motion metrics directly to the hypothalamus and the medial septum (line 569 – 585).*

-	My second main concern is about comparing two very different populations: LS fast spiking neurons and CA1 pyramidal cells. First of all, this point is not made clear enough in the text. Second, it is problematic for the analyses because:

*We agree with the reviewer that it poses certain challenges to compare inhibitory GABAergic neurons with CA1 and CA3 pyramidal cells. We have made changes to Fig 1C to better reflect what populations we are recording from, as well as indicating that we are imaging from GABAergic LS cells and pyramidal cells in dorsal CA1 and CA3 in the results section (line 116-118; 141) as well as in the figure legend (line 167-168). Additionally, we made changes in the introduction (line 92-92 ) to better indicate what the cell type is that we are comparing.*

*It is worth mentioning that as previous studies have shown, LS cells are not fast spiking (Gallagher 1995). To address the concerns of the reviewer, we have patched the LS GABAergic cells and provided measures of the intrinsic properties and firing frequency in GABAergic neurons of the lateral septum and compared those to parvalbumin-positive (PV) fast-spiking interneurons of the hippocampus (S5 Fig), confirming that the LS cells are not fast-spiking neurons. Additionally, we have provided measures of probability to be active in linear track (S6 Fig) and in open field (S12 Fig).*

o	1) Many CA1 pyramidal cells are silent or pseudo-silent in a given environment. This would influence many of the analyses presented here (e.g., modulation for a specific correlate, decoder). Reports of spatial/directional/self-motion coding on cells with insufficient activity rate are meaningless. Likewise, it is not right to compare decoders based on set of cells with different percentage of silent cells. The authors must set a threshold of activity (electrophysiologic studies have set such a threshold as 100 spikes/10 mins) for cells to be analyzed. ALL analyses, comparison of percentages and use of neurons for decoding must be re-done while considering only active cells (or comparable percentage of active cells).

*We acknowledge that low-frequency firing cells (pseudo-silent) may not contribute to spatial codes and could contribute to increased decoding error. While completely silent cells cannot be recorded using miniscopes because of the absence of fluorescence, our analytical method based on a comparison with shuffled surrogates was designed to identify cells that do not reject the null hypothesis that activity is spatially modulated. We are describing the reasoning behind our analytical approach in more detail in the results section (line 123-127) and refer to the new figure panels S4B,E Fig. We also provide a quantification of the P(A) versus fraction of neurons (S6B Fig).*

Additionally, t*o control for this potential confound, we performed the same analysis using a .001 activity probability, which corresponds to approximately 600 ms of calcium activity during periods of locomotor activity (>5m/s) in a 10 minute recording, and still replicated our original findings both in the 1D environment (S6D Fig) and in the 2D environment (S12 Fig). We also applied this cutoff to our Bayesian decoder for both 1D and 2D environments and recapitulated our original results (S8E Fig, S13F Fig).*

o	2) Even when comparing only active cells, LS fast spiking neurons have very different rate compared to CA1 or CA3 pyramidal neurons. The authors must check to which extend rate influence what they measure (e.g., scoring methods, decoder, etc.). If they observe a rate effect, they must control the effect on scoring methods by calculating "information per binarized unit of activity" or by downsampling. Likewise, they must control the effect on the decoder by proceeding to their random selection of cells to decode from on cells presenting similar rate or presenting downsampled rate when comparing CA1/CA3 and LS. In SF6, I noticed a higher number of spatial cells coding for area that are infrequently visited (centre). This raises concerns as to the fact that authors may include cells with too low threshold of activity.

*We agree with the reviewer that in spite of statistical control, activity levels might be correlated to spatial information, which could introduce a bias in our study given that we found LS cells to be more active than CA1 cells overall. As we observe a direct correlation between P(A) and MI (S4B Fig), in addition to controlling for this using our bootstrapping approach (S4C-E),we are now also providing a measure of MI (bits) per binarized event (Fig1H, Fig3C, Suppl 17A-C).*

*By selecting only cells with comparable firing or information properties, we would decode using a sample that constitutes an unfair representation of the structure of interest. Therefore, for our decoder, we are using an activity cut off of P(A) > .001 to only include cells that are above this threshold of activity (S8E Fig, S13F Fig). We are therefore confident that the results presented here cannot be explained by silent or pseudo-silent cells.*

-	My third main concern is that the number of mice in CA3 is too low to reach any conclusion. I think that for this result to be part of the main figures, the authors should present results from at least 3 mice. This is especially of concern given that I am suspecting that the imaging procedure may compromise the integrity of the hippocampal circuits by cutting intrahippocampal connective fibers. I would very much appreciate if the authors could ease these concerns by presenting complete histology of hippocampal recordings. The integrity of the hippocampal circuits is essential to assess spatial information there and to meaningfully compare it with LS spatial information.

*We agree with the reviewer that the number of CA3 animals included in this panel was low. Whereas originally we had n = 2 dCA3 animals for the linear track, we now have recorded from n = 6 animals, and for the open field experiments we used to have n = 5 dCA3 animals, where we have now included n = 8. We have added extra CA3 animals not only for this decoding panel (now Fig2D), but also for the 1D and 2D spatial and conjunctive coding analyses (main figure 1,2,3) and corresponding supplemental figures (S4 Fig, S6 Fig, S7 Fig, S8 Fig, S12 Fig, S13 Fig, S17 Fig).*

*We agree with the reviewer that it is important to the hippocampal circuits and the possible effects of this damage on the integrity of the spatial code. We have therefore provided the histology for the 6 available CA3 animals in S3A Fig. We also assessed the extent of the damage for CA3 implants along the anterior posterior axis (S3B Fig). Even though CA1 damage should not affect the CA3 representation, we have addressed this concern by comparing two different sets of CA3 implants, those that are implanted trans-hippocampally (with some damage to the CA1/2 region) and those implanted extrahippocampally. Specifically, we compared probability being active and mutual information of those place cells, and did not observe any difference (S3C Fig). We hope that this eases some of the reviewers concerns.*

-	My fourth main concern is that I have doubt about whether the authors considered the fact that CA1 pyramidal cells present a strong heterogeneity in spatial coding along the dorso-ventral and proximo-distal axis in CA1 (Henriksen et al 2010). I urge the authors to incorporate information on proximo-distal location of CA1 pyramidal cells when reporting analyses on spatial modulation. To my knowledge, only 2 examples of CA1 field of view are reported for now. I would like to see the others reported too. Likewise, injection in the CA1 for the tracing studies seems limited to the most distal portion of CA1 - an area which is known to have less spatial information. I would strongly advise the author to re-do tracing studies while targeting more proximal portion of the CA1 too.

*We thank the author for raising this important point. We agree that we need to provide information on proximal-distal location of CA1 pyramidal cells. To this end, we have now provided histology for CA1 implants in S2 Fig, in addition to the examples initially present in the main figures. In order to assess whether we observe any differences in mutual information along each axis, we color coded cells for each animal using their respective MI value in bits (S2B Fig). We then plotted MI value along either axis, and assessed whether position on the axis was correlated with MI (bits) (S2C Fig). We subsequently plotted the R^2^ values for both axes (S2D Fig). We did not find any significant modulation along any of those axes within our field of view.*

*For the tracing studies, we believe we are targeting a portion of CA1 that is also commonly targeted in electrophysiology studies. In the new version of the manuscript, we are providing a complete overview of the spread along the anterior-posterior axis of a representative animal (S19 Fig). This should help the reader to get a better idea of the spread of our injection, which infects cells at a moderate (not excessively proximal or distal) level. Unfortunately, targeting an even more proximal region would lead to leakage into the CA2/CA3 region that would bias our results.*

-	My fifth main concern is about the remapping analyses and is linked to an incomplete use -in my view- of the rich information provided by miniscopes. First: given the fact that the authors can follow the activity of individual cells across days, I do not understand why they estimate remapping based on the whole population. I would very much prefer to see a cell-by-cell analyses instead that would allow us to distinguish between 2 main scenarios for the LS population: 1) part of the population remain stable while the other remap and 2) all cells remap equally. Second: cell-by-cell analyses should allow the authors to address the crucial difference between rate and phase remapping. As stated above, we know that many CA1 cells are silent (or become more silent when the environment gets more familiar). Differencing between rate and phase remapping is thus essential to understand the dynamics between CA1 and LS.

*This cell-by-cell analysis unfortunately does not allow us to assess phase remapping, as miniscopes do not provide electrophysiological field potential information. Moreover, the lower temporal resolution of miniscopes (30 Hz) is incompatible with precise rate remapping analysis, which justified our probabilistic approach. Instead, Fig 4 focuses on analysis of global vs partial remapping.*

*Fig 4 has now been updated to address the important concerns of the reviewer. In addition to the individual examples shown in Fig 4B, we have expanded on the analysis and now show mean tuning map correlation for all cells across all day pairs (Fig 4C). We then show individual cells over days in panel 4D. We are seeing evidence for the first scenario proposed by the reviewer, with part of the population remapping whereas others stay stable. Additionally, we are now showing positive tuning map correlation vs proportion of cells in S13C Fig. Finally, we are now showing tuning map correlation comparisons for CA1 and LS for all day pairs (Fig 4F).*

Minor concerns:

-	It may be a matter of taste, but I am not sure that there is a consensus on the fact that directional information is purely idiothetic - according to me it is not.

*We agree with the reviewer. As directional information may not be purely idothetic, we have now rephrased certain key sentences to reflect this.*

*Abstract:* ‘...LS accurately and robustly represents spatial, directional as well as self-motion information…’ line 42-43)

Introduction ‘In addition to positional information, LS neurons may also encode direction and self-motion information, including acceleration and velocity’ (line 78)

Additional changes have been made in line 337, 424, 455, 467,

-	I would prefer if the authors could use the term spatially modulated cells rather than place cells. The definition of a place cell being narrower than what they present here.

*We agree that our analysis picks up on spatially modulated cells rather than place cells per se. We have made changes in the results, discussion, methods and figure legends to remove ‘place cell’ and replace it with ‘spatially modulated cell’ or ‘spatial cell’. Some mentions of the word ‘place cell’ remain in the introduction and discussion where deemed appropriate (such as when citing relevant literature or when discussing hippocampal properties)*

-	Careful when formulating that LS is the main subcortical output of the hippocampus. I don't think it is true when it comes to the subiculum or the dentate gyrus, even when limiting oneself to the dorsal portion. Also, the long-range GABAergic projection from CA1 to the MS should be mentioned already in the introduction. In general, hippocampal is used as a synonym for CA1-CA3 in the text. It should not.

*To the best of our knowledge, the LS was identified as the main subcortical output of CA1, CA3 and subicular pyramidal neurons (Swanson and Cowan, 1979; Risold and Swanson, 1997). This was quantified fairly recently by Tingley & Buzsaki (2018), who found that convergence of hippocampal efferents to LS neurons is orders of magnitude more dense 20-800 times than any of its cortical targets. In our updated manuscript, we have changed ‘main subcortical output’ to ‘major subcortical output’ (line 28) to avoid any issues..*

*We have now added a reference to the long-range GABAergic projection from CA1 to the MS in the introduction (line 57-58).*

*We have now removed mentions of ‘hippocampal’ and changed it to dorsal CA1 and dorsal CA3 where appropriate.*

-	References: L81: I would cite Swanson and Cowan, 1979; Risold and Swanson, 1997; L116: I would cite Muller and Kubie 1987. L159: Souza 2018 is in the text but not in the reference list.

*L81 (now line 74-75: We have now added Swanson and Cowan, 1979 in addition to Risiold & Swanson (1997) that was already present.*

*L116 (now line 108) This sentence is referring to spatial firing characteristics in the lateral septum. We do not feel that this citation is appropriate in that section, since Muller and Kubie (1987) studied the effect of changing the environment on spatial firing in the hippocampus. If there has been a mixup of line numbers, we are happy to add this citation to the section the reviewer suggests.*

*Souza 2018 is now in the reference list (line 1382), we thank the reviewer for suggesting this correction.*

-	L119 (and several times after): the authors make a point about the fact that: in contrast [to the hippocampal formation] LS has been implicated in behaviors that may require a stable representation of space over time, including context-reward, contextual fear conditioning and spatial learning and memory. This line of argument is flawed as a large body of studies have shown that the hippocampal formation is involved in all these behaviours. This argument is repeated in the discussion. This is problematic: CA1 (and the parahippocampal region) has been demonstrated to encode information about goal/reward.

*We agree with the reviewer that our initial formulation seemed to infer contrasting roles for CA1 vs LS in long-term encoding of space and reward, while our focus was to specifically investigate remapping properties in the LS. In our updated version, we are now stating that: 'While the LS, similar to the hippocampus, [LS has been implicated in representation of space over time....]. However, remapping dynamics in the LS remain largely understudied (line 84-87).*

-	Fig 1B, S1 (and subsequent histology/tracing images): I would very much prefer if number of infected cells were reported as estimated percentages rather than in raw numbers. As it is, the authors should report % infected cells/DAPI. However, it would be even better to proceed to additional Immunohistochemistry (IHC) staining to report percentages by cell type. For instance, to prove that they indeed target pyramids in the hippocampus. I am a bit puzzle by the use of rabies (a quite heavy procedure) to merely show projections from CA1-3 to the LS.

*We understand the need for normalizing our data. Unfortunately, using DAPI as a normalizing denominator would include many undesired cell types, including glial cells. Concerning immunohistochemical data, previous reports have shown that the hippocampal to LS projection arises from the pyramidal cells (Swanson, Sawchenko & Cowan, 1980 (not cited); Tingley & Buzsaki, 2018; Swanson & Cowan, 1979; Risold & Swanson, 1997), while inhibitory interneurons specifically project to the MS. We are now reporting the number of infected cells as the percentage of total infected cells, for each individual mouse .*

*Finally, we chose rabies as it enables a Cre dependent approach, which allows us to limit starter cells to only GABAergic cells in the LS, and assess pre-synaptic inputs in a monosynaptic fashion, as opposed to other options like CTB, fast blue, retrobeads, or AAV retrograde viruses. We have now added a sentence in the results section justifying the use of this technique (line 112-113).*

-	On S1: what is the percentage of starter cells? Could the authors please report the % of GFP+ only and mCherry+ only cells in the LS? That would give an idea of the efficiency of the rabies strategy. Did the authors verify that it actually infected only GABA+ cells and that there was no leakage?

*Unfortunately, the spread of GFP would just give us an impression of the total number of cells expressing TVA and oG (dependent only on injection volume). On the other hand, the spread of mCherry+ could give us an insight in local connectivity within the LS, but not inform on the efficiency of the rabies virus. While we are not drawing any conclusions on local connectivity, we do agree with the reviewer that we need to indicate the total number of starter cells. In the updated manuscript, we are now reporting the total number of starter cells for each animal individually for each LS subregion, in figure S1H.*

*The efficiency of the rabies strategy has been described by others, suggesting that this approach is relatively inefficient in terms of its ability to label input cells (for overview, see Callaway & Luo, 2015, not cited). As an example, Marshall et al., 2010 (not cited) expressed rabies into single starter pyramidal neurons in layer ⅔ of the mouse visual cortex and observed an average of 49 input cells per starter (these are cells with ~1200 dendritic spines, with an estimated ~600 inputs). Thus, we can assume that we are only labelling a very small proportion of the hippocampal inputs to LS, yet providing quantifications of mCherry+ and GFP+ cells will not give us an estimate of the efficiency.*

*Finally, the dorsal LS is well described to consist of GABAergic cells, with only a few glutamatergic cells found at the most ventral extremity (Lin et al., 2003). As we see in S1H Fig., we have not labelled any starter cells ventrally.*

-	Please use the terms infected and infection (or transduction) instead of transfected and transfection.

*We have changed all mentions of transfected or transfection and changed it to transduction where applicable.*

-	Fig 1B, S1: given that labelling is inequal depending on anatomical location, it is insufficient to only report the distance to the bregma if the plan is to spatially map inputs onto LS cells. It would be more accurate to take into account all parameters, including M/L, D/V and proximo-distal axis, as well as ideally cell type if further immunohistochemistry analyses are possible. Given that the results are pooled from 5 mice, I would appreciate if the authors could provide data/mouse on the graphs.

*We agree with the reviewer that additional anatomical information could benefit potential claims of specific hippocampal sub-circuit projects to the LS. Although highly interesting, the proposed approach is somewhat impossible: the spread of injected tracer is too large to start looking into precise ML/AP/DV axes specifics. As such, here we are not making specific claims concerning such projections, but propose to now report the number of infected cells as a percentage of total infected cells for each individual mouse to add information on potential discrepancies from mouse to mouse.*

-	L 171: "within-session stability of place cells was significantly different between LS, dCA1, and dCA3, stability in the LS was not significantly" Please reformulate.

*We apologize for the confusing formulation and have now rephrased this result (line 136-140).*

-	A few points on the decoder: 1) Do the result change without the temporal filtering of the decode position? The filtering introduces temporal correlations that could boost performance but not be related to the information content of the population. 2) The procedure for performance calculation is not illustrated clearly: the authors say they train on 50% of the data, but they do not say if the rest is used for testing, and how many times they do the train-test split. Please clarify. If it is not what the authors have already done, I would suggest a cross validation procedure (10-fold cross validation or leave-one-out cross validation are the most used in decoding approaches as far as I know). 3) Strong issue about rate (see main concern)

*1. We agree with the reviewer that the effect of temporal filtering must be controlled in our decoder. For linear track data, we have now added a control where we removed temporal filtering altogether (S8G Fig), and compared decoding error for cells recorded in LS, CA1 and CA3. While, decoding error is slightly decreased, it still is significantly lower than that of shuffled surrogates. Similarly, the observed differences between LS and CA3 remain when omitting temporal filtering (S8H Fig).*

*Complementary to this approach, for the open field decoding we are now showing the effect of different smoothing windows on decoding error in the LS (S13E Fig), where we see that with the absence of a temporal filtering, the decoding error gets slightly smaller, yet still remains significantly different between actual and shuffle. We believe that together, these added panels provide compelling evidence that our observed results are not due to performance boost induced by filtering.*

*2. We have now updated the methods section to describe the procedure more thoroughly. In short, using only epochs where the velocity of the animal was higher than 5 cm/s, we identified a training set using 50% of the data (random sampling). The remaining 50% of the data was used for testing. Decoding efficiency (as measured by a decoding error and a decoding agreement score as outlined below) was calculated using 30 bootstrapped surrogates using randomly chosen data points with replacement, to allow for computation of mean MI and SEM. In order to allow for fair comparison between recordings with different numbers of cells recorded, this approach was done using either 40, 60, 80 or 100 randomly chosen cells with replacement for each bootstrapped sample (S8 Fig, S13 Fig). This description is now given in line 858-862 of the method section.*

*3) As mentioned earlier in our response to the main concerns, the activity of LS cells is higher than that of CA1 cells. To control for these confounds, in addition to our statistical criterion based on a comparison with shuffled surrogates, we assessed the portion of place cells using arbitrary activity threshold corresponding to what has been typically used in electrophysiological studies (equivalent of a 1 Hz spiking threshold). We now control for the inclusion of cells with low activity, by using a threshold of P(A) > .001 (S8E,F Fig, S13F Fig).*

-	20 cm decoding error on a 100 cm track is not very accurate. I would adjust the text to reflect that. Maybe this is due to the inclusion of many silent cells in the CA1 assembly?

*Using calcium imaging, we cannot record silent cells since they do not generate calcium transients, and are hence never extracted when processing video recordings. For both linear track and open field, we have now added a panel with CA1 decoding using all cells, to show that the decoding error decreases significantly when using all of the available cells. The relatively large decoding error seen in the main figure is due to the low number of cells that we are including in the decoder (in order to provide a fair comparison between regions recorded with large 1.8 mm and small 0.5 mm GRIN lenses), and the fact that we train on 50% of a 10 minute recording session (while using a > 5 cm/s velocity threshold) using calcium transients. While these results might not directly compare to electrophysiological studies, random cell sampling was necessary to enable a fair comparison across brain regions. As mentioned above, we now control for the inclusion of cells with low activity, by using a threshold of P(A) > .001 (S8E,F Fig, S13F Fig.). Additionally, we have now adjusted the text accordingly: ‘To ensure the proper functioning of our decoder, we confirmed that the decoding error decreased significantly when increasing bootstrapped sample size (S8C,D Fig), line 192-194.*

-	3- Bursting index: I would suggest for clarity the probability of two consecutive active bins to be denoted with P(A,t|A,t-1) instead of P(A|A). A matter of taste, more than anything.

*We agree with the reviewer that this is a more appropriate indexing nomenclature. This has been changed both in the figure and in results section (line 236) and methods (line 802-803)*

-	Fig 1H and 3G are not informative with the present scales

*Fig 1H has been changed, and is now Fig 2A in the updated manuscript. Figure 3G has been updated and the scale has been changed.*

-	L240: no increase at food reward: not surprising in a novel environment. The degree of novelty/familiarity of the environment is improperly neglected in the analyses when this would have been very valuable information. When appropriate, please report number of exposures and time spent in each environment. Make sure to compare cells of animals receiving the same amount of exposure. This greatly influences spatial information content.

*We thank the reviewer for this comment. All animals received the same amount of exposure for these experiments, we have added a sentence in the methods section to clarify (line 707-709). For the food and object modulation figure, the experiment consisted of 5 recordings on consecutive days, with open field recordings in between recordings with food zones and objets. We have now added arrows to S10 Fig to better indicate this, and added explanation in method section line 710-715. We have quantified time spent in the food zone or object zone, and provided both total time spent as well as proportion of time spent (%) in S10 Fig.*

-	The difference in stability results between linear track and open field are not addressed.

*We agree with the reviewer that this point needs further discussion. One of the major feature of the linear track is the induction of directional place fields (for both CA1 and the LS). Since we show that LS cells integrate numerous allocentric as well as self-motion information, this could explain differences observed between linear track and open field stability. This point is now raised in line [488-490]*

-	Fig 3: 1) I would appreciate a picture evidencing virus infection in the LS. 2) Fig 3H "Tuning map correlation between day 1 and day 8" is based on only one animal for CA1. Is that the whole extends of the data? If so that seems insufficient to reach a conclusion on that point.

1) *We have now added a picture evidencing virus infection in the LS of an animal of this cohort as part of Figure 4A.*
2) *We have added 2 more CA1 animals to this experiment, to make a total of 3 CA1 animals included in this dataset, and compare those to 5 animals implanted in LS.*

Remapping analyses: I would have been curious to know more about partial remapping (near the border of the environment for example). The dataset presented here could be exploited to answer this interesting question. However, this analysis is not as important in view of the others.

*We agree with the reviewer that this question is interesting, and augmented our analysis of anchoring to objects/walls in S10 Fig. These examples do not suggest any anchoring to borders. Since our dataset will be publicly available, these questions will be open for anyone to explore beyond this study.*

-	Head direction (HD) analyses: is it head direction or heading? Please be more specific in methods. Also detail how velocity and acceleration are calculated. When showing polar plot for HD, please add a central circle to represent (hopefully) uniform head direction of the animal during that session. Also note in the discussion that retrosplenial cortex and parasubiculum also present large proportion of directionally modulated cells.

*We are referring to head direction, and not heading (which is an important distinction). Line 787-796 of the methods section now further details how we extract position, head direction, velocity and acceleration. Yet, as we have not done typical head direction experiments (stability of HD cells in the dark, assess remapping of HD cells in new environments), we proceeded by calling them ‘directionally modulated’ cells in the rest of the manuscript. In all polar plots indicating directional tuning, we have now added a central circle (blue) to indicate normalized head direction of the animal during that session (Fig 5A, G, S15 Fig, S16 Fig).*

*We have noted in the discussion that the retrosplenial cortex and parasubiculum also contain a large proportion of directionally modulated cells (line 538), and we thank the reviewer for pointing this out.*

-	Fig 5: As it is this figure is not fully supporting the authors claims. After reading the study that initially published the AAV1 virus used here, I could not see reports of any 2nd order neurons labelled. Therefore, I am not sure whether the virus can actually spread further than the 1st order neuron. Could the authors please discuss this point. This is problematic in light of S10 where the same technique is used to label direct inputs. It is not clear to me how they can claim that there is no second order labelling in S10. Furthermore, I am unsure that based on the evidence provided her, the author can reach the conclusion that there is direct disynaptic pathway from principal cells of the hippocampus to the medial septum. I also would like for the authors to show all the injected area rather than a few examples.

*We thank the reviewer for this comment. To summarize, the AAV1-Cre virus will infect neurons at the primary injection site. It then travels transsynaptically to the first order downstream neuron. In our study, we are using a Cre-dependent eYFP to visualize those first order neurons and their axonal projection pattern (presumably giving us an indication of the second order projection target), as is summarized in the schematic shown in Fig 6A. To prevent any confusion we have now removed the word second-order from the manuscript, both from the results section and from the figure legend of Fig 6, and is now replaced by ‘eYFP-positive axons’. In S19 Fig we are now showing all injected areas for both CA1 and CA3 primary injections. We have rephrased our results (line 398-391) on the direct disynaptic pathway, which is now: ‘we observed a direct pathway from the principal cells of the hippocampus, via the LS,leading to dense innervation of the medial septum’.*

-	It was not clear to me whether the same AAV1 Cre was used for 5D. Incidentally, I'm not sure that this is the best way to prove that these are synaptic connections. The method used her will indeed show that there are putative boutons in these areas, but to prove that these are synaptic connections, one need to do an IHC with another marker of the presynaptic terminal and a post-synaptic marker… Or to test this functionally with a ChR2 for instance. All in all, I would very much appreciate if the authors could highlight the novelty of their results here compared to what is already known on these projections. (also true for S9)

*We have now provided additional information on the injection strategy employed in Fig 6D (previously 5D) in the text line 400-403. We agree with the reviewer that this shows the existence of putative boutons, but that we cannot conclude that these are functional connections. We have therefore toned down our conclusion of this experiment (line 404) .*

*S9 is now S18 Fig, and is discussed in line 384-387.*

-	Fig 6 "thus it is likely that this information is directly inherited from the hippocampus." I do not think that the data presented here can support this statement. Inactivation studies would be more appropriate to test this.

*We agree with the reviewer that this statement could be toned down. We have now rewritten the final sentence of this paragraph as: This suggests that this information could be directly inherited from the hippocampus, which should be tested using targeted inactivation of the hippocampal pyramidal inputs to LS (line 453-454).*

Reviewer #4: PlosReview

The authors used 1-photon mini-scopes to record from neurons in the mouse lateral septum and assessed the coding characteristics of these neurons in terms of egocentric and allocentric spatial and behavioral parameters. In addition, they used transsynaptic viral tracing to characterize the efferent projection of LS neurons that receive direct hippocampal connections, showing connections with medial septal and hypothalamic regions not directly connected with hippocampus. The central finding is that LS neurons represents spatial, speed, and directional information in an anatomically-organized fashion in a manner corresponding somewhat to the mapping from D-V axis of hippocampus onto LS. The GABAergic LS spatial cells were quite comparable to hippocampal "place" cells.

Overall critique:

This is a very interesting paper that adds significantly to the literature on the inheritance, in extrahippocampal structures, of spatial correlates from the hippocampus. The technological achievement is laudable, and the analysis has been competently executed. I think the paper will be of interest to a wide audience. I have only quite minor comments and suggestions.

There are a few grammatical errors which can be left to the editors to correct.

"We found that even though within-session stability of place cells was significantly different between LS, dCA1, and dCA3, stability in the LS was not significantly different than in dorsal CA1 (Kruskall-Wallis, H(3) = 8.922, p = .0116, Figure 1G)." Sentence appears to be contradictory. Also, poor syntax: "different than" should be "different from".

*We have updated the stats for this sentence, and corrected this sentence (line 130). It now reads as: ‘ We found that within-session stability of spatially modulated cells (expressed as a correlation where 0 means no stability while 1 means perfectly stable) was significantly different between LS (0.44 ± .0165) and dCA3 (0.73 ± 0.26; Kruskal-Wallis, H(3) = 80.48, p < .0001; Dunn’s multiple comparisons test, p < .0001), and between dCA1 and dCA3 (p < .0001; Fig 1G)’ line 136-140.*

Figures: Text is generally too small, and the figures are mostly too complex. For example, in Figure 1 it would be better to split out A, B & C to a separate figure. Caption for Fig 1 lacks an explanation of part E - jumps from D to F

*We agree with the reviewer that the figures can be seen as too extensive. We have made several changes in the new version of the manuscript:*

- *We have split Figure 1 into two separate figures and placed them at the appropriate location in the results section. We have increased the size of the panels and the fonts used. We have adjusted the figure captions and made sure that an explanation of panel E was present.*
- *Figure 3 (previously figure 2) has been made bigger to improve visibility of the panels.*
- *Figure 4 (previously figure 3) has been made bigger, and some panels have been moved to S13 Fig.*
- *Figure 7C (previously 6C); fonts have been made bigger.*

*Supplemental figures have also been adapted where possible.*

"LS integrates direction, velocity, and acceleration information" - This is perhaps a bit of an overstatement. LS cells express such information, but it is not clear in what sense they integrate it, nor is it even clear what that would mean. Integration of velocity gives speed, integration of speed gives relative position, but I don't know what integrating direction would give.

*We agree with the reviewers point. We have now changed the term ‘integrate’ to ‘represent’ in line 326.*

Care needs to be taken in interpreting 'directional modulation' in open field situations where walls restrict access of the animals to certain orientations in certain positions.

*We agree with the author that it is important to take into account the restrictions that come with recording directional modulation in a square open field. In all polar plots indicating directional tuning, we have now added a central circle (blue) to indicate normalized head direction of the animal during that session and found a relatively uniform distribution of orientations (Fig 5A,G, S15 Fig, S16 Fig).*

Authors might wish to point out that this is not the only case of GABAergic neurons inheriting some spatial specificity from hippocampal pyramidal cells. Indeed, interneurons within CA1 show a very comparable effect - higher firing rate, greater spatial dispersion but overall comparable information per second (Maurer et al., 2006, J. Neurosci. 26:13485).

*This is a very valuable point. We are now including this information in our discussion section, line 525.*

Although it seems pretty likely that the observed correlates are indeed inherited from hippocampus, the authors should at least address the possibility that it comes from other brain regions, that project to LS, of which there are many.

*We agree with the reviewer and are now pointing this out in our discussion, line 758 - 761.*

References to the literature on the change in the properties of hippocampal cells along the DV axis would be appropriate in the discussion section (Jung et al., J. Nrsc. 1994, 14, 7347; Kjelstrup et al., Science 2008, 321, 140).

*We agree with the reviewer that this is an essential point to add to our manuscript. We have now added the missing references and updated our discussion (line 513-517).*

Typo: "The question arises of what the function of could be having a region downstream" - move the second "of" to after "be".

*The typo has been corrected (line 548).*

"Thus, through this process of coincidence detection and recurrent inhibition, the spatial map could be compressed to be represented by a much lower number of cells. As such, the LS could accurately convey hippocampal information to downstream regions using a much sparser signal, thereby allowing for increased metabolic efficiency (Olshausen and Field, 2004) and more effective associative learning (Isely et al., 520 2010; Palm and Sommer, 2006; Zetsche). " … It's a matter of opinion perhaps, but I think that this section is a bit incorrectly phrased. The idea of compression appears to be correct and has been brought up previously in the context of hippocampal projection to subiculum, for example. But compression means that the output code is actually more 'distributed' not sparser, i.e., in LS, a greater proportion of the neurons is activated at a given location, the firing rates are higher, and so the net information per second transferred is the same. Such a non-sparse

code is more appropriate for data transmission rather than associative storage. There is increased metabolic demand at the single cell level, but considerably fewer cells can be used to transmit the same information.

*We agree with the reviewer and apologize for the misuse of the term 'sparse', and instead refer to 'distributed' place fields. We have updated the manuscript (line 562 to line 565). We thank the reviewer for the kind comments and highly relevant suggestions, as well as the time spent reviewing our manuscript.*
